# Functional analysis of LFRFamide signaling in Pacific abalone, *Haliotis discus hannai*

**DOI:** 10.1371/journal.pone.0267039

**Published:** 2022-05-05

**Authors:** Sungwoo Yoon, Mi Ae Kim, Jung Sick Lee, Young Chang Sohn

**Affiliations:** 1 Department of Marine Bioscience, Gangneung-Wonju National University, Gangneung, Gangwon-do, Republic of Korea; 2 East Coast Life Sciences Institute, Gangneung-Wonju National University, Gangneung, Gangwon, Republic of Korea; 3 Department of Aqualife Medicine, Chonnam National University, Gwangju, Jeonnam, Republic of Korea; Universite de Rouen, FRANCE

## Abstract

The invertebrate LFRFamide (LFRFa) and short neuropeptide F (sNPF), consisting of 6 to 10 amino acids, are orthologs for bilaterian NPF/Y, which consist of 36 to 40 amino acids. Recently, a molluscan G protein-coupled receptor (GPCR) for NPF was characterized in Pacific abalone (*Haliotis discus hannai*). To address the functional evolutionary route of the invertebrate LFRFa and NPF signaling system, in this study, we identified cDNAs encoding LFRFa precursors and the sNPF receptor (Hdh-sNPFR) in Pacific abalone. Four LFRFa mature peptides with 6 or 7 amino acids were predicted: GSLFRFa, GGLFRFa, GTLFRFa, and GSTLFRFa. Hdh-sNPFR was identified as a classical rhodopsin-like GPCR and classified into a molluscan sNPFR group. In HEK293 cells, Hdh-sNPFR was mainly localized in the cell membranes and internalized in the cytoplasm following treatment with LFRFa peptides. Reporter assays demonstrated that LFRFa peptides inhibit forskolin-stimulated cAMP accumulation in Hdh-sNPFR-expressing HEK293 cells. *LFRFa* precursor and *Hdh-sNPFR* transcripts were more strongly expressed in the cerebral and pleural-pedal ganglia of Pacific abalone than in the peripheral tissues such as the ovary, gills, intestine, and hepatopancreas. The levels of *LFRFa* transcripts in the ovary, intestine, and hepatopancreas were significantly higher in mature female abalone than in immature females. Injection of LFRFa induced the egg release and spawning behavior of mature abalone, but suppressed food intake. These results suggest that LFRFa peptides are endogenous ligands for Hdh-sNPFR involved in food intake and reproduction through a Gαi-protein dependent signaling pathway.

## Introduction

The Gastropoda are the largest and most diverse class of the phylum Mollusca (i.e., accounting for approximately 80% of all mollusks), exhibiting the highest diversity in molecular physiology, morphology, and ecology [1]. In gastropods, neuropeptides such as FMRFa-like peptides (FLPs) are expressed early and potentially play important roles during molluscan development, generating a broad diversity of FMRFa in spatially restricted patterns in the nervous system of abalone [2]. Abalone (Mollusca: Vetigastropoda: Haliotidae) has been regarded as one of the most commercially important mollusks worldwide, and there has been a rapid increase in the farmed production of abalone, especially in China and South Korea, which has helped to compensate for the worldwide demand and exploitation by overfishing to some extent [3]. Recently, the abalone aquaculture industry in South Korea has been facing the challenge of high mortality during the seed culture period along with slow growth traits of abalone species. To overcome these problems, projects to develop breeding programs for genetic improvement and environmental adaptation have been established in South Korea [4]. In previous studies, we explored neuropeptides and their signaling systems in the Pacific abalone, *Haliotis discus hannai*, through transcriptome and proteome analyses [5, 6]. Recently, we revealed the presence of the orexigenic neuropeptide F (NPF) signaling system in Pacific abalone [7].

Neuropeptide precursors are synthesized in the nervous system of animals and are then further processed in the secretory vesicles, where they are stored until secreted by exocytosis in response to external or internal cues and are then transported to the peripheral organs where they regulate physiological processes and behavior in diverse animal phyla. Neuropeptide molecules have been explored in several invertebrate species by genomic and proteomic approaches as important model organisms for human diseases, disease vectors, and pest species [8–12]. Similar to vertebrate neuropeptides, invertebrate neuropeptides play diverse roles in controlling physiological processes and adaptive animal behaviors such as feeding/metabolism, reproduction, development, ecdysis, circadian rhythms, and sensorimotor integration [13–15].

Secreted neuropeptides bind to and activate specific G-protein coupled receptors (GPCRs) on target cells to modulate neural and hormonal activity, leading to changes in the activity of downstream effector proteins such as enzymes and ion channels [16]. Recent advances in comparative transcriptomics/genomics have enabled the discovery of uncharacterized neuropeptides and their endogenous GPCRs in diverse invertebrate taxa, including deuterostomian echinoderms [17, 18], providing new opportunities to gain insights into the evolution of neuropeptide signaling pathways in the Bilateria. For example, sequence analysis of the *Drosophila melanogaster* genome identified at least 44 genes encoding putative GPCRs for neuropeptides, with many being orthologous to pharmacologically functional neuropeptide receptors in vertebrates [8, 10].

Since identification of the cardioexcitatory neuropeptide FMRFamide (FMRFa) from the clam *Macrocallista nimbosa* [19], structurally similar FLPs have been detected in animals of all major phyla [20]. In mollusks, FLPs exert pleiotropic activities and mediate a variety of physiological and behavioral processes [21]. FLPs that display varying sizes but harbor the common C-terminal RFa sequence can be divided into five distinct groups in mollusks: FMRFa, LFRFamide (LFRFa), luqin, NPF/Y, and cholecystokinin/sulfakinin (CCK/SK)-related peptides [21]. Interestingly, analyses of sequence and gene structure indicated that molluscan LFRFa is more closely related to the short NPF (sNPF) family in invertebrates [22, 23]. Since the first sNPF was isolated from the American cockroach *Periplaneta americana* [24], sNPF family members have been identified in a broad range of arthropod taxa, especially in insects and crustaceans [20, 25]. The invertebrate sNPF peptides typically consist of 4 to 11 amino acids (aa), including the evolutionarily conserved C-terminal M/T/L/FRF/Y/Wamide motif, whereas the so-called “long NPF/Y” displays a C-terminal RXRFamide motif with a length ranging from 36 to 40 aa across bilaterian species [26]. Although both sNPF and NPF/Y display C-terminal sequence similarities and have common roles such as the coordination of feeding across bilaterian species, they are likely evolutionarily distant. There is evidence that sNPF and NPF/Y arose as separate signaling systems in the common ancestor of deuterostomes and protostomes [26], and the sNPF system appears to be protostomian-specific [27]. Adding to this complexity, a phylogenetic and chromosomal analysis revealed a close relationship between prolactin-releasing peptide (PrRP) receptors and NPY receptor families in vertebrates [28], whereas the sNPF/PrRP-type signaling system is orthologous to the lost NPF/Y-type signaling systems in the Echinodermata, a deuterostome invertebrate phylum [23]. sNPF peptides play important roles in a variety of physiological processes such as in the regulation of feeding and growth [29, 30], metabolic stress [31], locomotion [32], and vitellogenesis and sexual maturation [33]. In the Pacific oyster *Crassostrea gigas*, LFRFa was demonstrated to serve as an endogenous ligand for an sNPF-type GPCR and the function of oyster LFRFa in the regulation of food intake is similar to that of insect sNPFs [22].

The first sNPF receptor (sNPFR) was characterized in *D*. *melanogaster* [34] and subsequently in diverse insects and marine invertebrates, including Pacific oyster [22, 26]. Insect sNPFRs showed concentration-dependent inhibition of forskolin-stimulated cAMP accumulation and/or a dose-dependent calcium response in Chinese hamster ovary (CHO)-K1 and human embryonic kidney (HEK) 293T cells [34–36]. Pacific oyster LFRFa peptides, but not FMRFa or NPF, could activate the oyster sNPFR and induced intracellular calcium mobilization in HEK293T cells, which suggested coupling of the oyster sNPFR to the Gq protein-mediated protein kinase C (PKC) pathway [22]. However, to the best of our knowledge, the Pacific oyster sNPFR is the sole functional receptor for LFRFa peptides identified in mollusks to date; thus, further characterization of LFRFa and sNPFRs is required to fully understand the LFRFa signaling system in mollusks.

Since the NPF and sNPF signaling systems were suggested to have branched off from their common ancestor early in evolution, prior to the split of the deuterostome and protostome lineages [26], investigation of the sNPF-like signaling system in mollusks is needed to understand the full evolutionary history and regulatory pathways of the sNPF system in bilaterians. Here, we report the LFRFa signaling pathway with ligand-specific sNPFR in Pacific abalone, demonstrating its potential involvement in the control of feeding, energy metabolism, and reproduction. These findings can provide new context and targets for improving the productivity, conservation, and aquaculture of abalone.

## Materials and methods

### Sequence analyses of abalone LFRFa precursor and sNPFR

Nucleotide sequences for the *H*. *discus hannai* (Hdh) LFRFa precursor (NCBI GenBank accession number OL804262) and sNPFR (GenBank accession number OL907301) were identified in previously reported transcriptome databases [5, 6]. The nucleotide sequences were compared with those in public databases, including the National Center for Biotechnology Information (NCBI) BLAST programs. The aa sequence alignments for representative LFRFa/sNPF-related peptides (S1 Fig, S1 Table) were performed using Clustal Omega Multiple Sequence Alignment with default parameters [37]. The aa sequence alignment and prediction of transmembrane helices for Hdh-sNPFR were performed using CLC Genomics Workbench software (CLC Bio, Aarhus, Denmark) and the latest version of the TMHMM program [38], respectively. The BoxShade program (https://embnet.vital-it.ch/software/BOX_form.html) was used to highlight conserved aa sequences. The N-linked glycosylation and intracellular phosphorylation sites were predicted by the NetNGlyc and NetPhos servers, respectively (https://services.healthtech.dtu.dk/). To generate phylogenetic trees, aa sequences of invertebrate LFRFa/sNPF-related precursors (S1 Table) and sNPF/NPF/NPY-related receptors (S2 Table) were retrieved from the literature [7, 23, 39, 40] and the NCBI databases. In total, 29 neuropeptide precursors and 95 receptors were aligned and automatically trimmed as described previously [7]. The trimming contained a total of 54 and 215 residues for neuropeptide precursors and receptors, respectively, which were used to generate the maximum-likelihood phylogenetic tree with W-IQ server v1.6.12 [41]. The substitution models, VT+G4 for neuropeptide precursors and mtInv+F+I+G4 for neuropeptide receptors, and the ultrafast bootstrap approximation approach with SH-aLRT 1000 replicates were used. Phylogenetic trees were visualized using the free software package FigTree v1.4.3.

### cDNA cloning and plasmid construction of abalone sNPFR

A three-year-old female Pacific abalone (7.7 cm shell length; 59.2 g body weight, BW) was purchased from a local dealer (Yangyang, Gangwon-do, Korea). Total RNA was extracted from the neural ganglia tissues, cerebral ganglia (CG), and pleuro-pedal ganglia (PPG) using the RNeasy Mini kit (Qiagen, Valencia, CA, USA). To obtain full-length *Hdh-sNPFR* cDNA, first-strand cDNA was synthesized using a SMART rapid amplification of cDNA ends (RACE) kit according to the manufacturer’s instructions (Clontech, Palo Alto, CA, USA). Polymerase chain reaction (PCR) was performed using the neural ganglia cDNA as a template and oligo primer sets (Table 1). For 5′-RACE and 3′-RACE of *Hdh-sNPFR*, the first-strand cDNA was used as a template for PCR amplification with Universal Primer Mix and a gene-specific primer set (Table 1; Clontech). Both 5′- and 3′-RACE PCR were performed in a 50-μL reaction volume using Advantage 2 Polymerase Mix (Clontech). The cycling conditions for 5′-RACE were as follows: 2 min at 95°C; 35 cycles of 20 s at 95°C, 40 s at 64°C, and 40 s at 72°C; and 5 min at 72°C. The conditions for 3′-RACE were as follows: 3 min at 94°C; 35 cycles of 20 s at 94°C, 40 s at 66°C, and 40 s at 72°C; and 5 min at 72°C. The PCR-amplified products were cloned into the pGEM-T Easy vector (Promega, Madison, WI, USA), which was transformed into *Escherichia coli* DH5α competent cells for amplification and sequencing. To construct the Hdh-sNPFR-expressing plasmid, the full-length cDNA encoding Hdh-sNPFR was PCR-amplified with oligo primers (Table 1), the neural ganglia cDNAs, and Vent DNA polymerase (New England Biolabs, Ipswich, MA, USA) according to the manufacturer’s instructions. The cycling condition was as follows: 2 min at 95°C; 35 cycles of 30 s at 95°C, 30 s at 58°C, and 80 s at 72°C; and 5 min at 72°C. The PCR-amplified products were digested by EcoRI and XbaI, and cloned into the restriction enzyme sites of the hemagglutinin (HA)-pcDNA3 expression plasmid (Invitrogen, Waltham, MA, USA). The plasmid constructs were analyzed to verify the correct sequence by Sanger sequencing.

**Table 1:** Oligo primer sequences used in polymerase chain reaction.

Target	Direction	Sequence (5ʹ–3ʹ)	Application
*Hdh-sNPFR*	Sense	GGTGACCAATAAGACGGACAGCTACGCATGC	RACE-PCR and cDNA cloning
*Hdh-sNPFR*	Antisense	TCCAGTTCATCCCGCTCCCGCGTCC	
*Hdh-sNPFR*	Sense	CGCGAATTCATGTCTCTTATTACGTCATCC	
*Hdh-sNPFR*	Antisense	GCGTCTAGATCACGTGTCATCAACTCTATTG	
*Hdh-sNPFR*	Antisense	TTGACGCGGAAACGTGCAAGGTG	
*prepro-LFRFa*	Sense	TCTATCCTCATGCTGGTTTTCG	quantitative PCR
*prepro-LFRFa*	Antisense	CACGTTTGTCCATGTCATAAGC	
*Hdh-sNPFR*	Sense	ACAAGCCCCCCGTTATGAG	
*Hdh-sNPFR*	Antisense	ATGCCCAGGAGGAAGATGATAC	
*RPL5*	Sense	TCACCAACAAGGACATCATTTGTC	
*RPL5*	Antisense	CAGGAGGAGTCCAGTGCAGTATG	

*Note*. Underlines indicate restriction enzyme recognition sites.

### Peptide synthesis

Mature peptide sequences from the Hdh-LFRFa precursor were predicted by the SignalP-5.0 (http://www.cbs.dtu.dk/services-/SignalP) and NeuroPred (http://stagbeetle.animal.uiuc.edu/cgi-bin/neuropred.py) servers along with previous alignment data for mollusk LFRF sequences [21, 22, 42]. Peptides for Hdh-LFRFa, Hdh-NPF, and RFamide (RFa) were custom-synthesized by Anygen Co., Ltd. (Gwangju, Korea) with a purity of >95% analyzed by high-performance liquid chromatography (Table 2).

**Table 2:** Amino acid sequences of peptides.

Peptide name	Sequence	Molecular weight (g/mol)	Purity (%)
GSLFRFa	GSLFRF-NH_2_	724.9	98.9
GGLFRFa	GGLFRF-NH_2_	694.8	99.4
GTLFRFa	GTLFRF-NH_2_	738.9	99.1
GSTLFRFa	GSTLFRF-NH_2_	826.0	99.1
RFa	RF-NH_2_	320.4	98.1
Hdh-NPF	QDAMLAPPDRPSEFRSPDQLRQYLKALNEYYAIVGRPRF-NH_2_	4609.2	95.0

### Cell culture and reporter assay

HEK293 cells were grown in monolayer culture in Dulbecco’s modified Eagle medium (Gibco, Loughborough, UK) with 10% fetal bovine serum (HyClone, GE Healthcare, Chicago, IL, USA) and 1% penicillin/streptomycin (Invitrogen, Carlsbad, CA, USA) at 37°C, 5% CO_2_. HEK293 cells were seeded in 24-well plates and the transfection was performed using a formulated polyethylenimine solution (Sigma-Aldrich, St. Louis, MO, USA) as previously described [43]. The Hdh-sNPFR expression plasmid in HA-pcDNA3 or HA-pcDNA3 alone (100 ng), luciferase reporter plasmids containing the cAMP response element (CRE-Luc) or serum response element (SRE-Luc) (100 ng), and the Rous sarcoma virus-β galactosidase expression plasmid (100 ng, internal control) were co-transfected into HEK293 cells as previously described [43]. At approximately 36 h post-transfection, the cells were maintained in fetal bovine serum-free Dulbecco’s modified Eagle medium for starvation for a further 16 h. The cells were then treated with Hdh-LFRFa peptides, RFa, or NPF; forskolin (Sigma-Aldrich); 12-O-tetradecanoylphorbol-13-acetate (Sigma-Aldrich); or the same volume of peptide-free medium as a vehicle for 6 h. The cells were harvested with a cell lysis buffer (Promega, Madison, WI, USA) and luciferase activities were assayed using a microplate luminometer (Berthold, Bad Wildbad, Germany) and normalized by β-galactosidase values detected on a microplate reader (Tecan, Männedorf, Switzerland) at 405 nm.

### Immunocytochemistry and confocal microscopy

To detect Hdh-sNPFR expression, HEK293 cells were seeded on poly-d-lysine hydrobromide (Sigma-Aldrich)-coated coverslips in 24-well plates. The Hdh-sNPFR expression plasmid was transfected into HEK293 cells as described above. At approximately 30 h post-transfection, the cells were treated with LFRFa peptides and NPF for 5 min and 30 min, respectively. The cells were fixed with 4% paraformaldehyde for 10 min, followed by treatment with 1% bovine serum albumin in phosphate-buffered saline with 0.1% Tween 20 (PBST) for 30 min at room temperature to block non-specific binding. The HEK293 cells were treated with a monoclonal HA primary antibody (1:5000 dilution; H9658, Sigma-Aldrich) in PBST at 4°C for 16 h. After washing with PBST three times for 10 min each, the cells were treated with a secondary antibody [anti-mouse IgG (H+L), F(ab’)2Fragment (AlexaFluor 488 Conjugate), 1:2000 dilution; Cell Signaling, Danvers, MA, USA] in PBST at room temperature for 1 h in a light-blocked chamber. After washing with PBST three times for 10 min each, the cells were mounted with a mounting medium including DAPI (Abcam, Cambridge, UK). The expression of Hdh-sNPFR was monitored with a confocal laser-scanning microscope (FV3000, Olympus, Tokyo, Japan).

### Detection of abalone *LFRFa* precursor and *sNPFR* transcripts

Pacific abalone (shell length, 8.7 ± 0.1 cm; BW, 73.9 ± 0.9 g; n = 33) were purchased from a local dealer (Gangneung, Gangwon-do, Korea). The neural tissues (CG and PPG), ovary, gills, intestine, and hepatopancreas were dissected, rapidly frozen in liquid nitrogen, and stored at –80℃ before RNA extraction. The maturity of the ovary was histologically examined according to a previous study [44]. The total RNA of each tissue was extracted using the RNeasy Mini kit (Qiagen) and 1 μg of RNA was reverse-transcribed to first-strand cDNA using the PrimeScript RT reagent kit with gDNA Eraser (Takara, Osaka, Japan). Quantitative PCR was performed with template cDNAs (10 ng), oligo primers for target and reference mRNAs (10 μM each; Table 1), and SYBR Premix Ex-Taq on Applied Biosystems 7500 Real-Time PCR System (Applied Biosystems, Foster City, CA, USA) using the following reaction conditions: 50°C for 2 min, 95°C for 10 min, followed by 40 cycles of 95°C for 15 s and 60°C for 1 min. We tested four candidate reference genes for this analysis, ribosomal protein L5 (*RPL5*), cyclophilin (*CY*), ubiquitin-conjunction enzyme (*UBC*), and elongation factor 1-alpha (*ELF*), according to the stability assessed with several statistical methods: the coefficient of variation, regression coefficient, and efficiency of amplification [45]. The most stable gene was determined to be *RPL5*, which was therefore used as the reference gene for quantitative PCR (S3 Table). The relative mRNA expression levels were calculated according to the formula: 2^–(Ct target gene–Ct reference gene)^. All results are expressed as the mean ± standard error of the mean.

### LFRFa injection and food intake

In August 2020, Pacific abalone (26.0 ± 5.1 g BW, n = 40) were purchased from a local dealer (Gangneung, Gangwon-do, Korea), kept in a flow-through seawater aquarium (21 ± 1°C; 400 L) for one week, and fed *ad libitum* on kelp (*Saccharina japonica*) before use in experiments. The abalone were starved for two days, placed in individual containers, and refed for one day before the experiment. On the day of the experiment, kelp pieces were divided into two equal parts, blotted, and weighed to obtain the wet mass (g) before and after the feeding period as previously reported [7]. Abalone were weighed prior to each assay (n = 8 per group) and 350 μL of mollusk saline (13 g HEPES, 25.66 g NaCl, 0.82 g KCl, 1.69 g CaCl_2_, 10.17 g MgCl_2_, 2.56 g Na_2_SO_4_, 1.0 L dH_2_O; pH 7.2) including LFRFa peptides or RFa (2.5 μg/g BW) was injected into the adduct muscle sinus using a 26-gauge needle. Control abalone were injected with the same volume of mollusk saline alone. Injected abalone were individually placed in a cage (15.5 × 11 × 6.5 cm) with flow-through seawater and supplied with seawater-immersed kelp equivalent to 7% of the BW. Food intake was assessed at 16 h post-injection as follows: Consumption (W) = [Wi × (WCf/WCi)–Wf], where Wi is the initial wet kelp weight, Wf is the remaining wet kelp weight, and WC is an autogenic control to determine the permeation of water into kelp during the feeding time. Consumption values were standardized for abalone BW to 100 g.

### LFRFa injection and spawning

In August 2020, mature female abalone (84.9 ± 5.2 g BW, n = 50) were kindly provided from an aquaculture farm (Wando, Jeollanam-do, Korea), kept in a flow-through seawater aquarium (23 ± 1°C; 400 L) for one week, and fed *ad libitum* on kelp before use in the experiment. On the day of the experiment, the abalone were randomly distributed into four groups (n = 7 per group) and 100 μL of mollusk saline including LFRFa peptides, RFa (2.5 μg/g BW), or the same volume of saline alone was injected into the adduct muscle sinus using a 26-gauge needle. Injected abalone were individually placed in a plastic tank filled with 2 L of filtered seawater with aeration. At 2 h post-injection, the spawned eggs were collected using a Muller gauze strainer, suspended in 30 mL of seawater, and the number of eggs recovered was counted using a stereomicroscope (SKX53; Olympus, Tokyo, Japan) according to the dilution factor.

In May 2021, mature female and male abalone (shell length 72.9 ± 6.0 mm, BW ~80 g; n = 60 for each sex) were prepared for spawning. The abalone were randomly distributed into five groups (n = 12 per group) and 100 μL of mollusk saline including LFRFa peptides (0.3 or 3.0 μg/g BW) or the same volume of saline alone was injected into the adduct muscle sinus. Injected abalone were individually placed in a plastic tank (2 L) and the frequency of spawning was measured for 1 h based on the spasmodic vertical motion as previously reported [46].

### Statistical analysis

Statistical analyses were performed with SPSS v.25.0 software (SPSS Inc., Chicago, IL, USA) or SigmaPlot 12.3 (Systat, Inc., San Jose, CA, USA). Statistical significance was determined with one-way analysis of variance followed by Bonferroni’s multiple test or Student’s *t* test, when data had a normal distribution. In some cases, data were log-transformed prior to analysis to meet the parametric assumptions of normality and equal variance. When data were not normally distributed, the nonparametric Mann-Whitney *U* test was used.

## Results

### Sequence analysis of abalone LFRFa precursor

**Fig 1:**
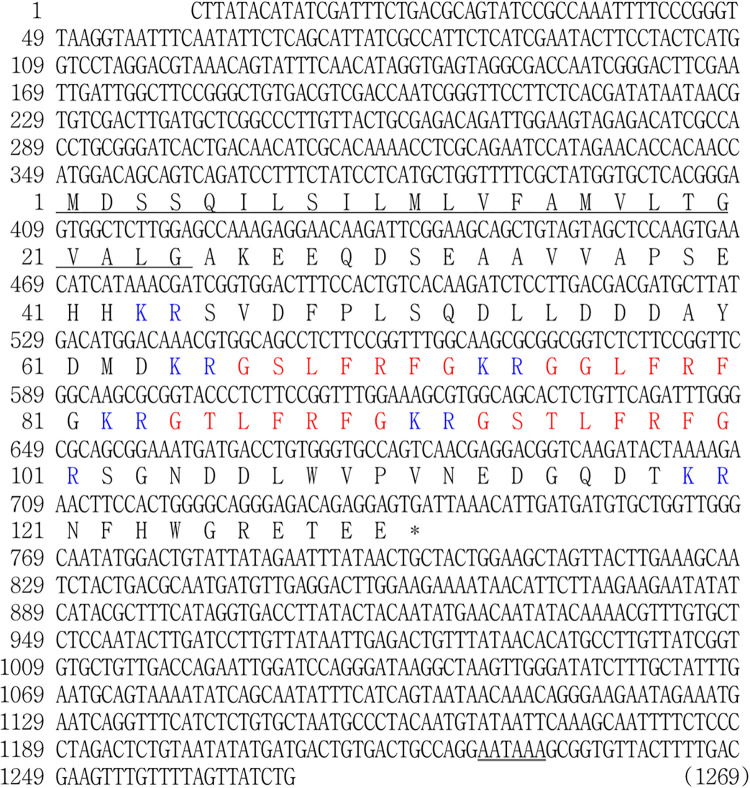
Nucleotide and amino acid sequences of *Haliotis discus hannai* LFRFamide (Hdh-LFRFa) precursor. Nucleotides and amino acids of Hdh-LFRFa precursor are numbered on the left. The predicted signal peptide sequence, mature Hdh-LFRFa peptides, and dibasic cleavage site residues are shown in underlined, red, and blue letters, respectively. The stop codon and polyadenylation signal are denoted by an asterisk and a double-underline, respectively. The nucleotide sequence for Hdh-LFRFa precursor has been deposited in the NCBI GenBank database (accession no. OL804262).

A putative cDNA encoding the Hdh-LFRFa precursor was identified, which was 1269 base pairs (bp) long, and included a 348-bp 5′-untranslated region, 393-bp coding sequence (CDS), and 528-bp 3′-untranslated region. The CDS of the Hdh-LFRFa precursor contained four types of LFRFa peptides: GSLFRFa, GGLFRFa, GTLFRFa, and GSTLFRFa. The cleavage sites were identified at the C-terminus of LFRFa sequences and a glycine residue allowing the amidation of the peptide was identified (Fig 1).

**Fig 2:**
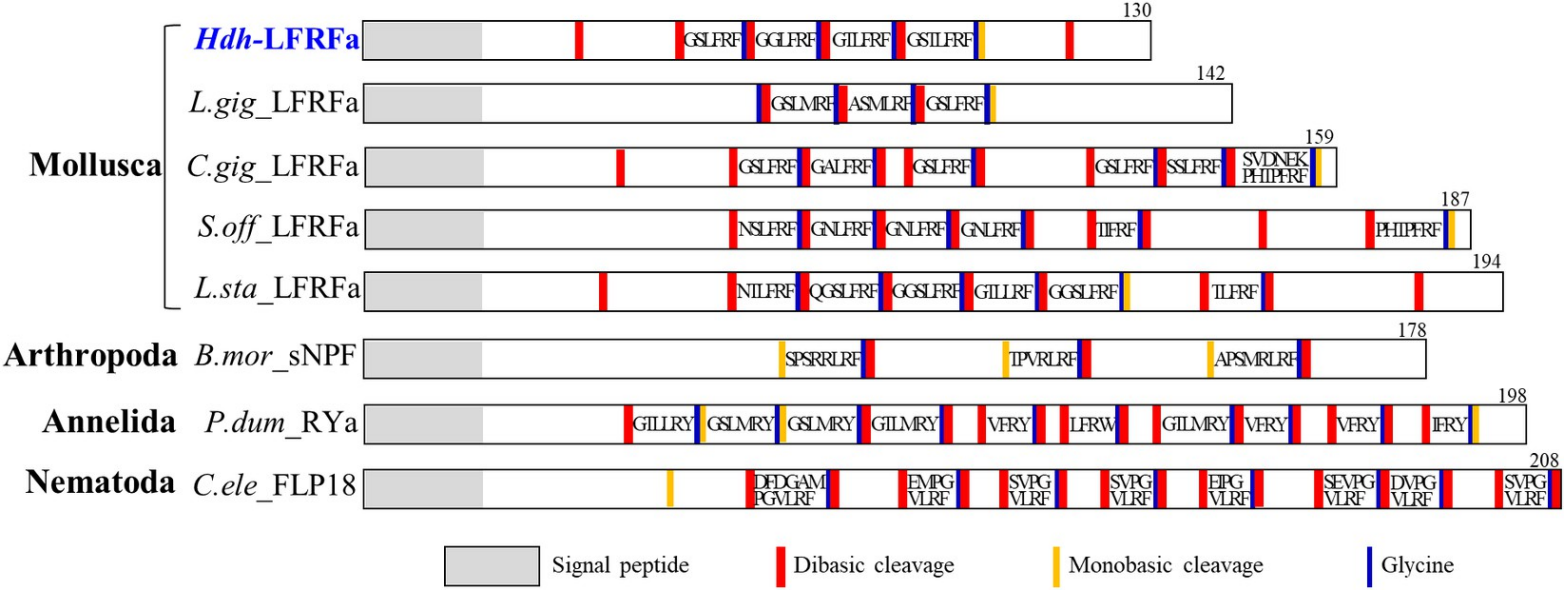
Amino acid sequence analysis of *Haliotis discus hannai* LFRFamide (Hdh-LFRFa) precursor. Comparison of the linear schematic organization of LFRFa/sNPF-related precursors in invertebrates. Signal peptides, proteolytic processing sites, and C-terminal glycines for amidation are indicated by distinct labels.

The molluscan LFRFa precursors contained one of several types of LFRFa peptides in *H*. *discus hannai*, *Crassostrea gigas* (Pacific oyster), *Lottia gigantean*, *Lymnaea stagnalis*, and *Sepia officinalis*, although the aa lengths of the CDS varied among species from 130 to 194 (Fig 2). The maximum-likelihood phylogenetic tree with invertebrate LFRFa, sNPF, and sNPF-related precursors, along with two molluscan APGWa precursors as an outgroup, showed that the aa sequence of the Hdh-LFRFa precursor grouped with those of the molluscan LFRFa precursors into a lophotrochozoan LFRFa/RYa/NPP subfamily, which was distinct from the arthropod sNPF and NPF/Y subfamilies (S1 Fig). Multiple sequence alignment analysis of mature LFRFa and sNPF peptides showed high C-terminal homology in diverse phyla (S2 Fig). Interestingly, the molluscan LFRFa retained almost the complete C-terminal LFRFa sequences, whereas other invertebrate sNPF and sNPF-related peptides showed the canonical RF/Y/Wamide C-terminal sequence only.

### Sequence analysis of Hdh-sNPFR

**Fig 3:**
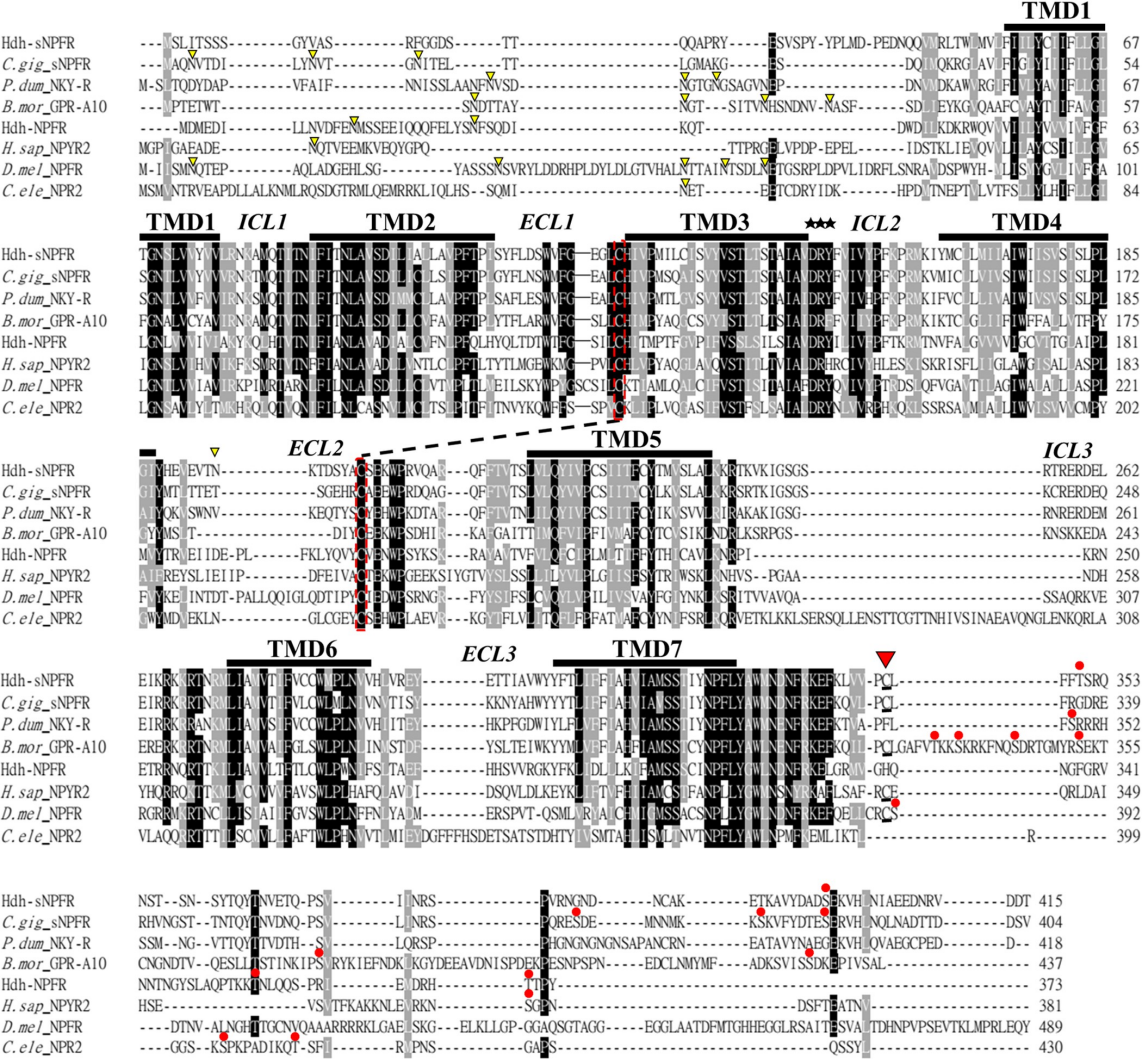
Amino acid sequence alignment of sNPFR-related receptors. The predicted seven transmembrane domains (TMD1–7) with three extracellular and intracellular loops (ECL1–3 and ICL1–3) are indicated on the aligned sequences. Identical and highly conserved residues (>70%) are shaded in black and gray, respectively. Potential N-linked glycosylation sites, the characteristic E/DRY/F sequence of rhodopsin-like GPCR, and consensus PKC and PKA phosphorylation sites are denoted with yellow arrowheads, black stars, and red dots on the amino acids, respectively. A putative disulfide bridge connecting ECL1 and ECL2 and a presumed palmitoylation-linked Cys-residue in the C-terminal intracellular domain are indicated by a dotted line and red arrowhead, respectively. Sequence abbreviations are listed in S2 Table.

**Fig 4:**
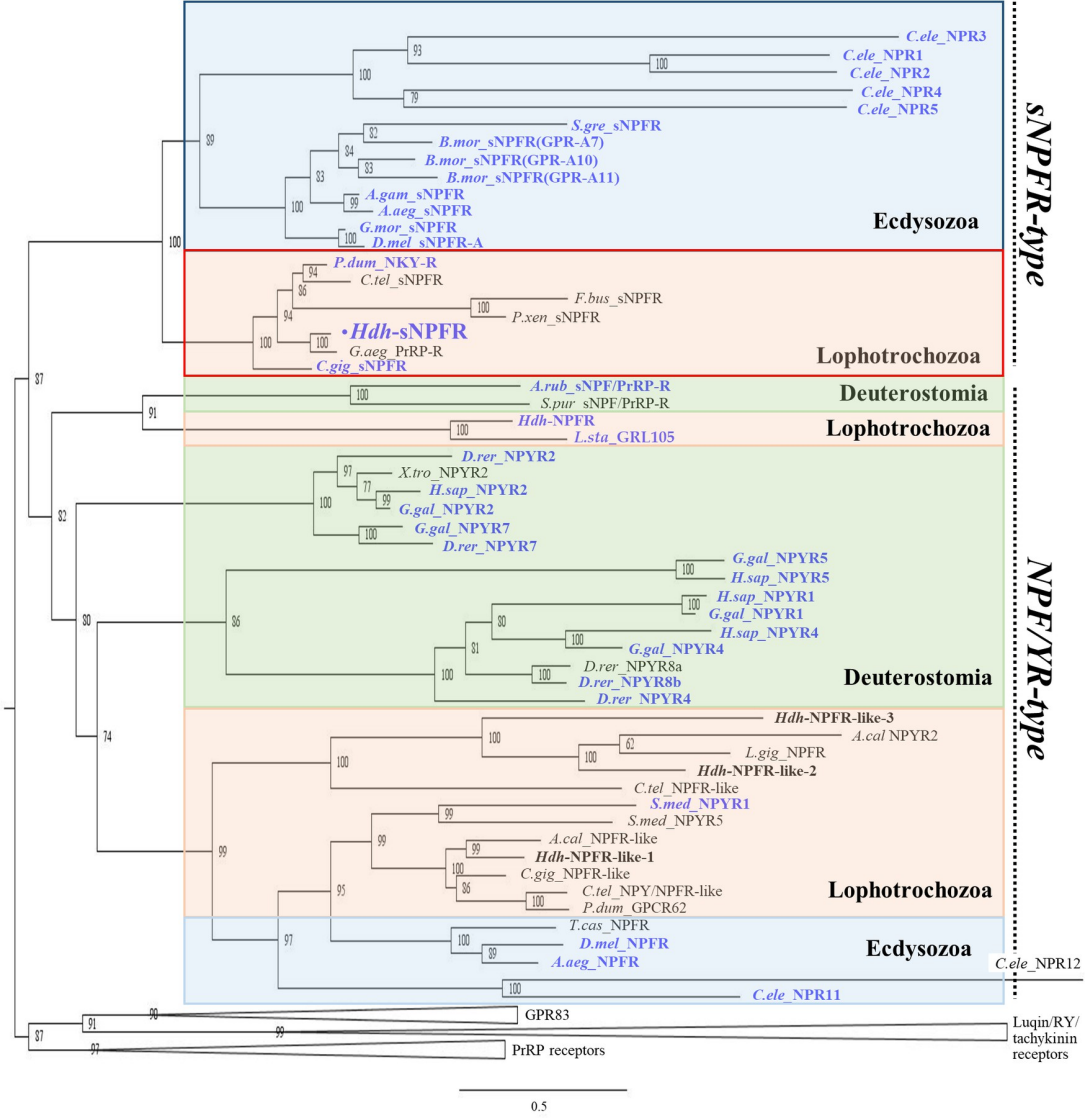
Phylogenetic tree of Hdh-sNPFR with protostome sNPF-type, bilaterian NPF/Y-type, and deuterostome PrRP-type receptors. Luqin/RYamide-type, tachykinin-type, and GPR83-type receptors were used as outgroups (condensed). Amino acid sequences of the receptors (see S2 Table) were aligned and the 215 trimmed residues were used to generate the maximum-likelihood tree using W-IQ server. Ultrafast bootstrap values are given at each branch, and the scale bar indicates amino acid substitutions per site. Deorphanized receptors for which receptor–ligand interactions have been experimentally characterized are colored in blue.

An abalone *Hdh-sNPFR* cDNA encoding a 415-aa-long protein was identified by a BLAST search with sNPFRs from the Pacific oyster *C*. *gigas* and the silkworm *Bombyx mori* (Fig 3). The identified Hdh-sNPFR showed a typical GPCR structure with one N-terminal extracellular domain, seven transmembrane domains (TMDs), three extracellular loops (ECLs) and three intracellular loops (ICLs), and one C-terminal intracellular domain (ICD). The characteristic E/DRY/F sequence of rhodopsin-like GPCR was detected in the second ICL (ICL2) and one potential N-glycosylation site was identified in the second ECL (ECL2) of Hdh-sNPFR. In addition, a disulfide bridge between the two C-residues connecting the ECL1 and ECL2, and a palmitoylated C-residue in the ICD were observed, which is consistent with the structure of most rhodopsin-like GPCRs [47]. Two consensus PKC phosphorylation sequences (R/K-X-S/T) were present in the ICD of Hdh-sNPFR, consistent with the consensus PKC and PKA phosphorylation sites (R-X-S/T or R-R/K-X-S/T) in the ICD of the orthologous *C*. *gigas* sNPFR, *B*. *mori* GPR-A10, and *Platynereis dumerili* NKY receptor. Phylogenetic analysis revealed that Hdh-sNPFR is positioned in a clade comprising protostomian sNPFR family members, which is distinct from a clade comprising larger bilaterian NPF/Y receptors (Fig 4). More specifically, Hdh-sNPFR was nested in the subclade composed of the deorphanized *C*. *gigas* sNPFR and the marine annelid *P*. *dumerili* NKY receptor.

### Luciferase reporter assays of Hdh-sNPFR in HEK293 cells

**Fig 5:**
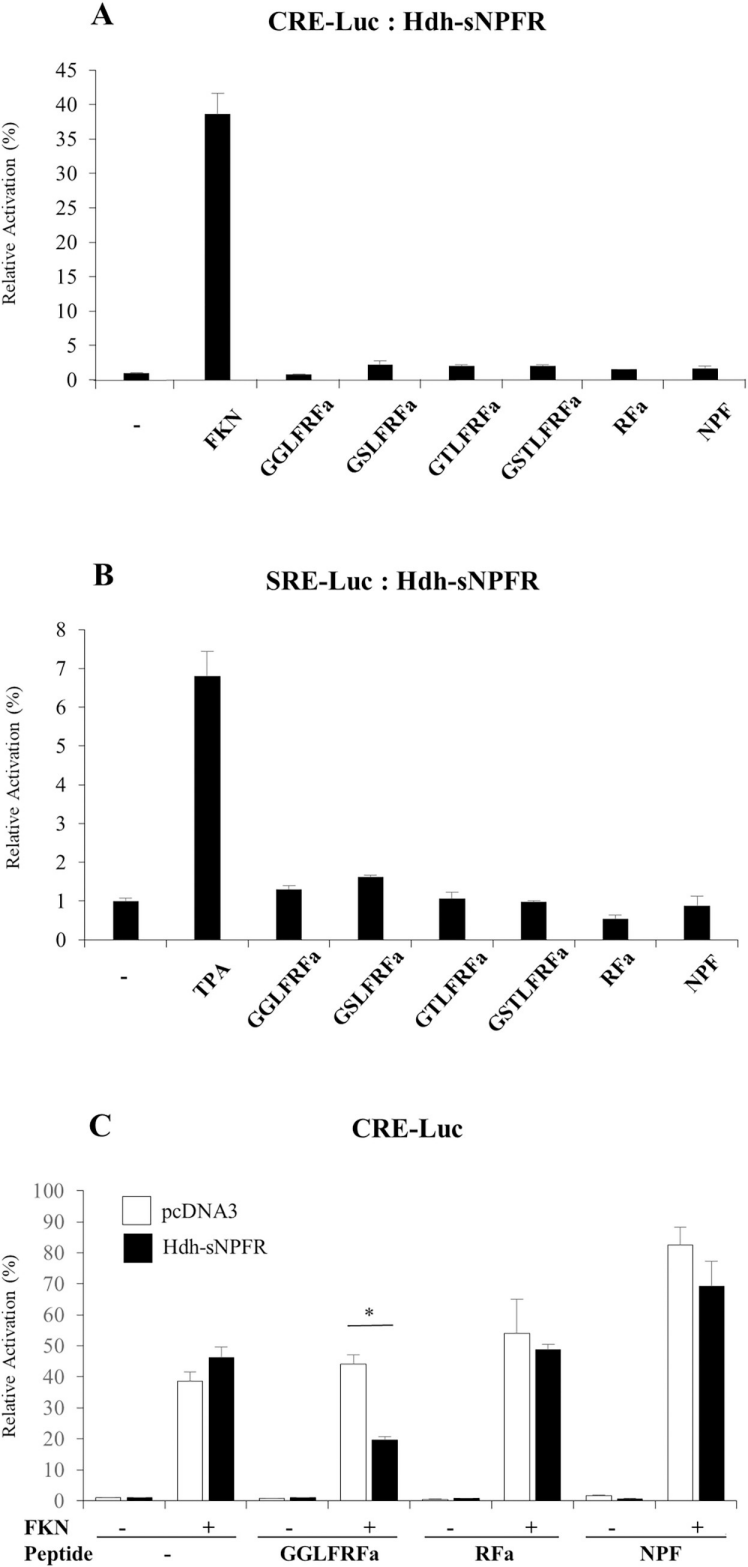
Effect of LFRFa peptides on luciferase reporter activities in Hdh-sNPFR-expressing HEK293 cells. **(A)** cAMP response element luciferase (CRE-Luc) reporter and **(B)** serum response element (SRE-Luc) reporter activities. HEK293 cells were transiently transfected with the Hdh-sNPFR expression plasmid in combination with CRE- or SRE-Luc reporter plasmids, and pRSV-β galactosidase plasmid as the internal control. Approximately 36 h post-transfection, the cells were maintained in serum-free medium for starvation for 16 h and treated with the indicated peptides (10^−6^ M), forskolin (FKN; 10^−5^ M), or 12-O-tetradecanoylphorbol-13-acetate (TPA; 10^−7^ M) for 6 h. **(C)** Effect of GGLFRFa, RFa, and NPF on FKN-stimulated CRE-Luc activities. Intracellular cAMP accumulation was measured by CRE-Luc reporter activities in Hdh-sNPFR- or maternal plasmid pcDNA3-transfected HEK293 cells. The relative activities were determined in response to 10^−5^ M of FKN and 10^−6^ M of peptide as indicated. All data represent the mean ± SEM (n = 3); *p < 0.05.

**Fig 6:**
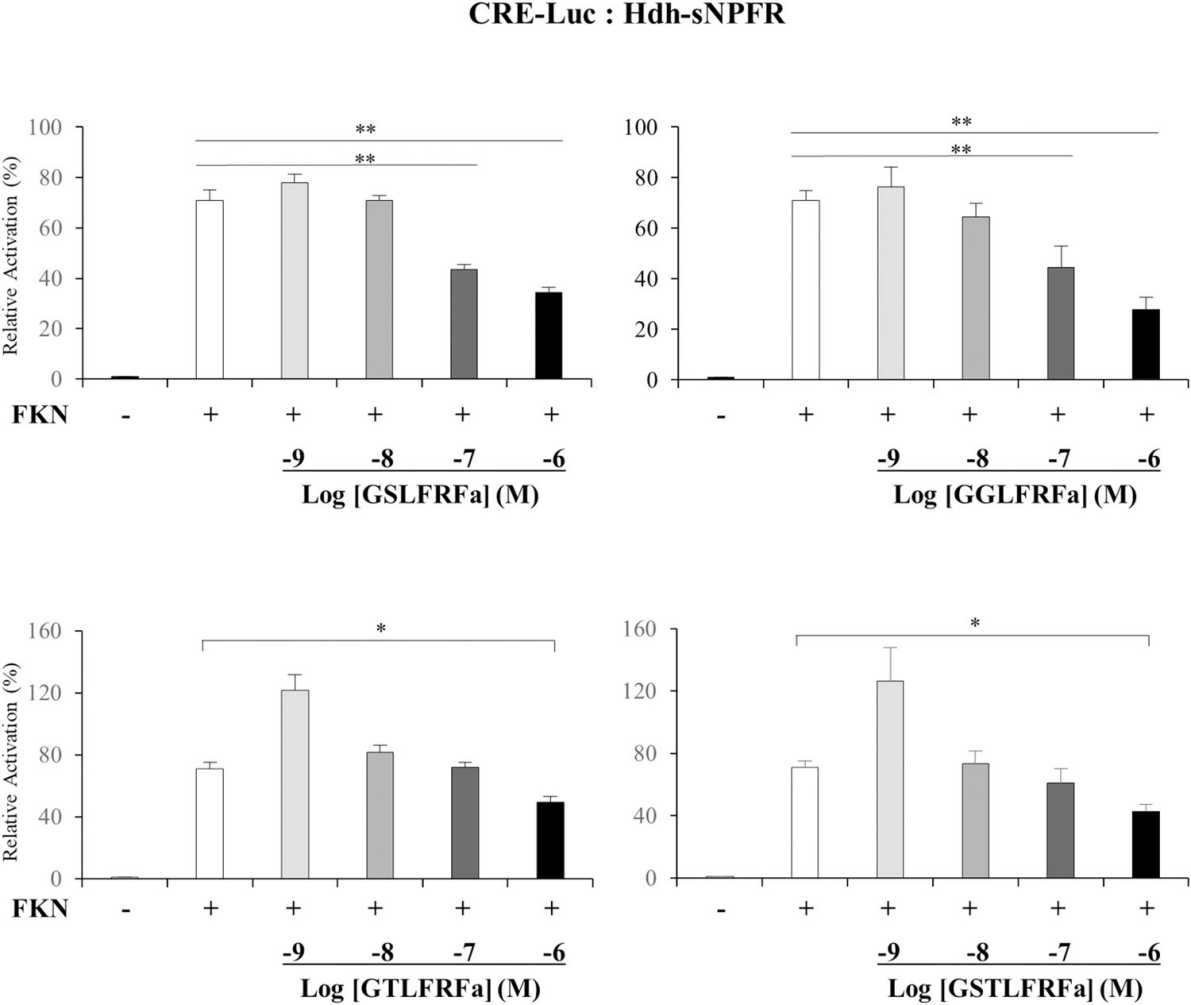
Effect of Hdh-LFRFa peptides on forskolin (FKN)-stimulated CRE-Luc activities in Hdh-sNPFR-expressing HEK293 cells. HEK293 cells were transiently transfected with Hdh-sNPFR, CRE-Luc reporter, and pRSV-β-galactosidase plasmids, and treated with FKN (10^−5^ M) and various doses of LFRFa peptides (10^−9^–10^−6^ M), as described in the legend to Fig 5. All data represent the mean ± SEM (n = 3); *p < 0.05, **p < 0.01.

To determine the signaling pathways involved in the Hdh-sNPFR, luciferase reporter systems under control of a minimal promoter containing CRE or SRE were applied in Hdh-sNPFR-transfected HEK293 cells. The synthesized Hdh-LFRFa peptides, RFa, and NPF (10^−6^ M each) did not activate CRE-Luc and SRE-Luc reporters in the HEK293 cells (Fig 5A and 5B). However, Hdh-LFRFa peptides, but not RFa and NPF, significantly (p < 0.05) inhibited forskolin-stimulated CRE-Luc activity in the HEK293 cells in dose-dependent manners (Figs 5C and 6). The 50% inhibitory concentrations of GGLFRFa and GSLFRFa were estimated as 3.2 × 10^−8^ M and 1.0 × 10^−8^ M, respectively.

### Cellular localization of Hdh-sNPFR in HEK293 cells

**Fig 7:**
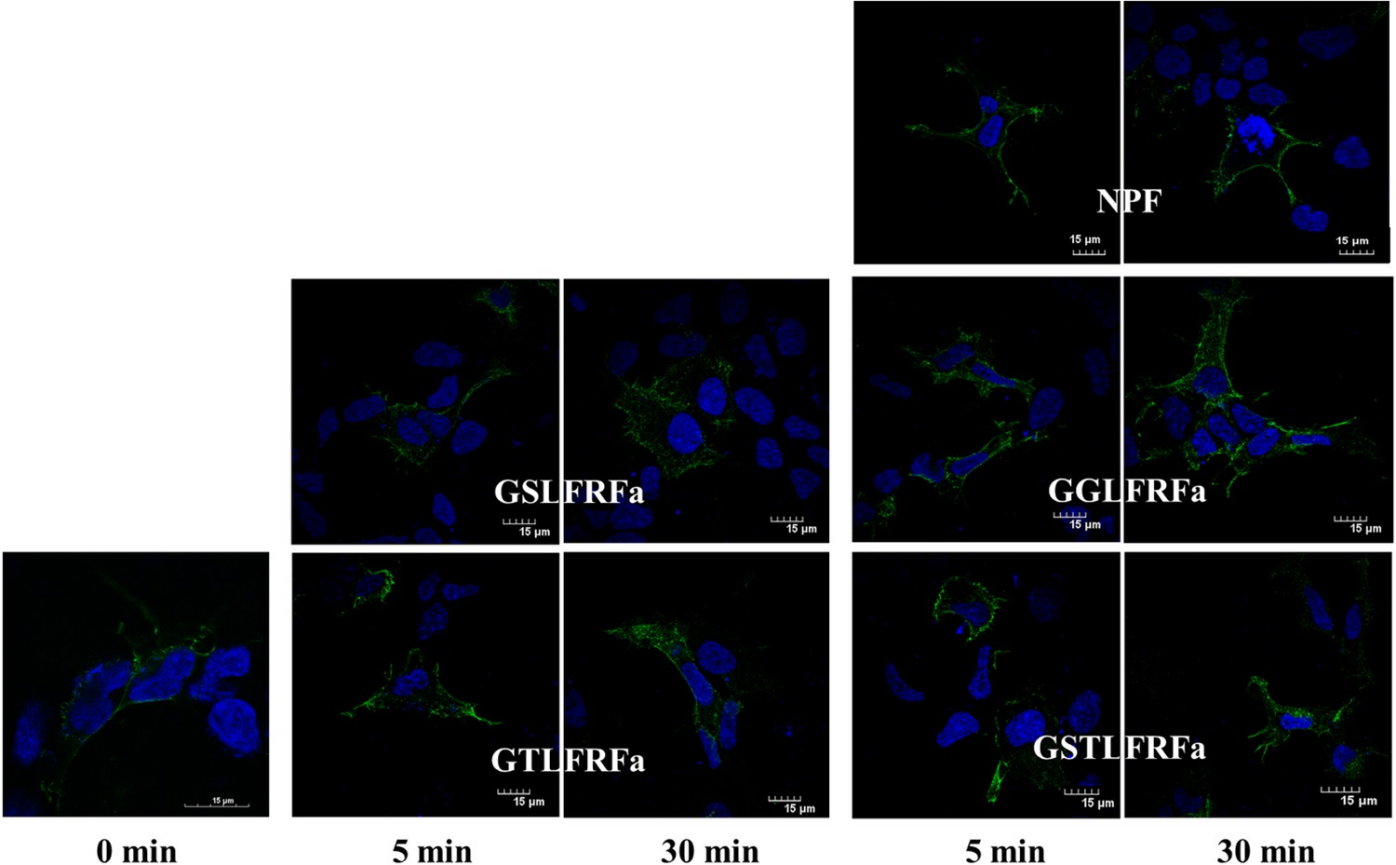
Subcellular localization and internalization of Hdh-sNPFR. HEK293 cells were transiently transfected with Hdh-sNPFR plasmid and treated with LFRFa peptides or NPF (10^−6^ M) for 5 and 30 min, respectively. HA-tagged Hdh-sNPFR was probed with a primary antibody directed against HA and labeled with an Alexa Fluor 488-conjugated secondary antibody (green); nuclei were counterstained with DAPI (blue). Scale bars = 15 μm.

To confirm the functionality of Hdh-sNPFR as a membrane receptor, HA-tagged Hdh-sNPFR was transiently expressed in HEK293 cells. Owing to the difficulty in obtaining available cell lines derived from abalone tissues, we investigated the membrane localization and membrane dynamics of Hdh-sNPFR heterologously expressed in HEK-293 cells. Cell surface expression of Hdh-sNPFR was observed using fluorescent confocal microscopy (Fig 7). The potency of Hdh-LFRFa peptides to induce the internalization of Hdh-sNPFR was determined by immunocytochemistry. Upon stimulation with Hdh-LFRFa peptides for 5 and 30 min, Hdh-sNPFR on the cell membrane moved into the cytoplasm, providing evidence for the interaction of Hdh-LFRFa peptides with Hdh-sNPFR (Fig 7). However, upon treatment with the NPF, the Hdh-sNPFR did not undergo redistribution from the cell surface to the cytoplasm.

### Tissue distribution of *Hdh-LFRFa* precursor and *Hdh-sNPFR* transcripts

**Fig 8:**
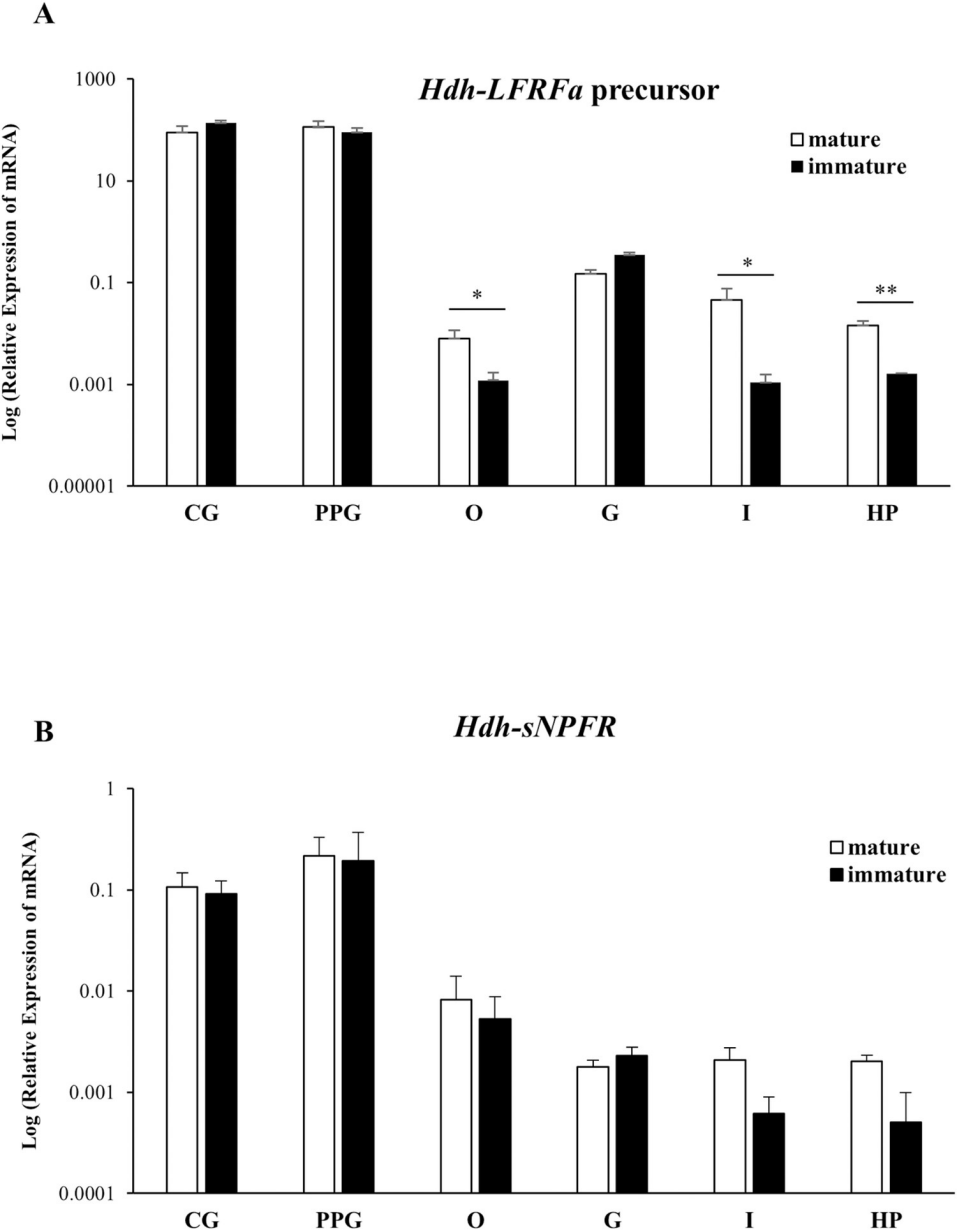
Relative expression levels of *Hdh-LFRFa* precursor and *Hdh-sNPFR* transcripts in various tissues of mature and immature female abalone. **(A)** *Hdh-LFRFa* precursor and **(B)** *Hdh-sNPFR* mRNAs were quantified by quantitative PCR and normalized in the log scale. All data represent mean ± SEM (n = 5); *p < 0.05, **p < 0.01. CG: cerebral ganglion; PPG: pleuro-pedal ganglion; O: ovary; G: gills; I: intestine; HP: hepatopancreas.

*Prepro-Hdh-LFRFa* and *Hdh-sNPFR* mRNA expression patterns were investigated in the neural ganglia (CG and PPG) and in various tissues of mature and immature female Pacific abalone. *Prepro-Hdh-LFRFa* and *Hdh-sNPFR* transcripts showed markedly higher levels in the CG and PPG compared with those in the ovary, gills, intestine, and hepatopancreas (Fig 8A and 8B). The *prepro-Hdh-LFRFa* transcript levels of the ovary, intestine, and hepatopancreas were significantly higher in mature abalone than those in immature animals (p < 0.05). No significantly different levels of *Hdh-sNPFR* transcripts were detected between mature and immature animals in all tissues examined (Fig 8B).

### Effect of Hdh-LFRFa peptides on food intake

**Fig 9:**
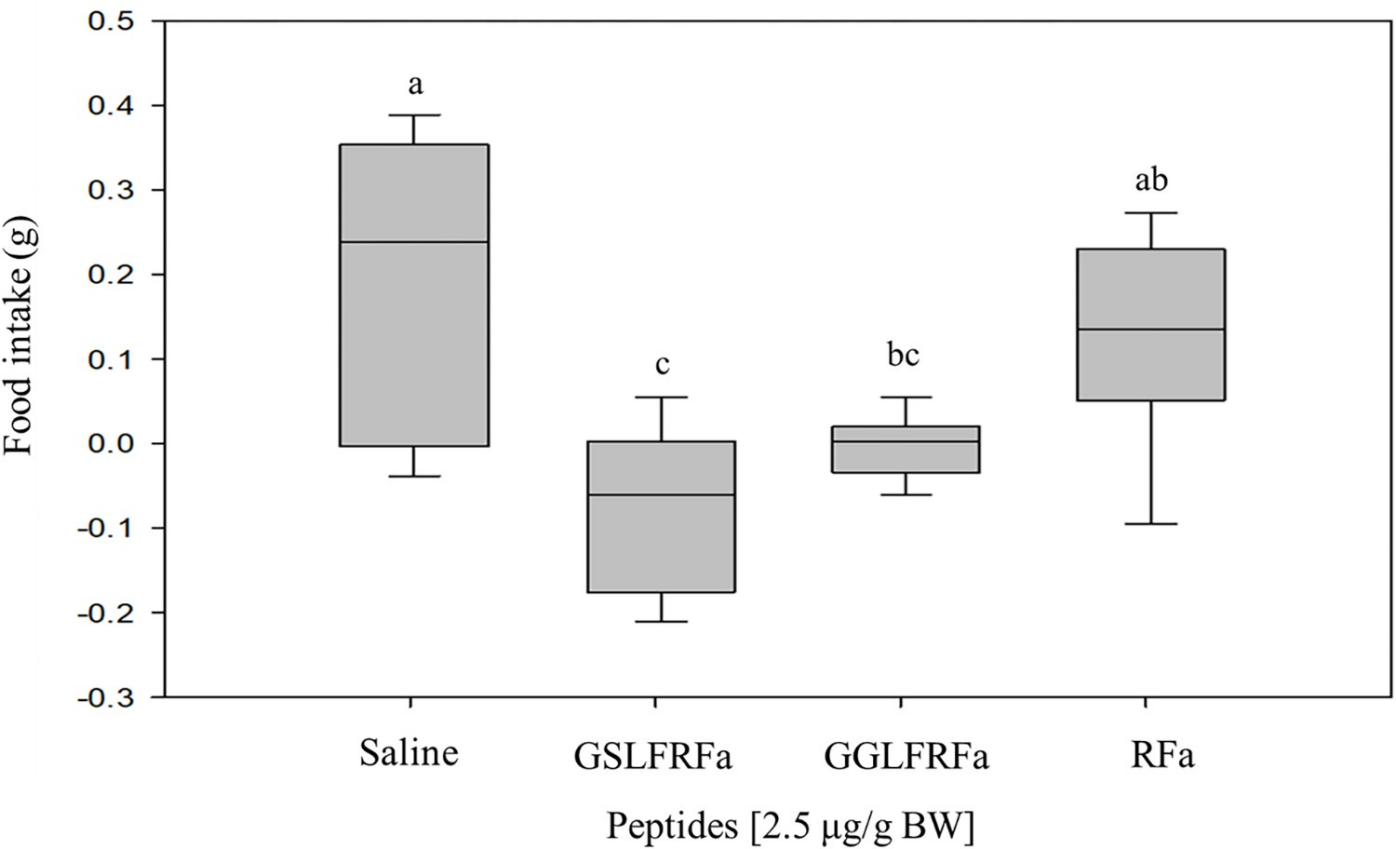
Effect of Hdh-LFRFa peptides on abalone food intake. Abalone were injected with GSLFRFa, GGLFRFa, RFa (2.5 μg/g BW), or the same volume of mollusk saline. Feeding assays were performed 16 h after injection. Data are presented as mean ± SEM (n = 8). Different letters indicate statistically significant differences (p < 0.05).

To investigate whether Hdh-LFRFa peptides affect food intake, GSLFRFa, GGLFRFa, and RFa (2.5 μg/g BW each peptide) were injected into adult abalone and kelp consumption was measured for 16 h. Food intake of abalone was significantly (p < 0.05) suppressed by GSLFRFa and GGLFRFa, but not by RFa, when compared with that of abalone injected with mollusk saline as a control (Fig 9).

### Effect of Hdh-LFRFa peptides on spawning

**Fig 10:**
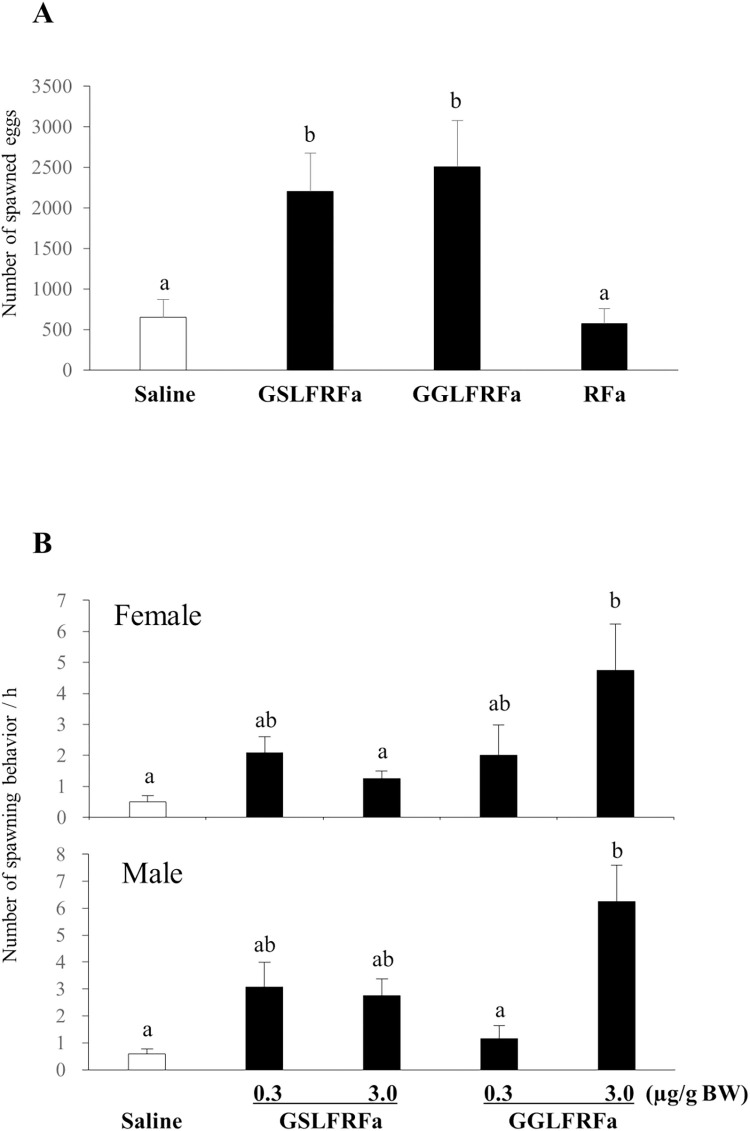
Effect of Hdh-LFRFa peptides on spawning in mature abalone. (A) Female abalone were injected with GSLFRFa, GGLFRFa, RFa (2.5 μg/g BW per peptide), or the same volume of mollusk saline (n = 7 per group). The number of spawned eggs was counted at 2 h post-injection. (B) Female and male abalone were injected with GSLFRFa, GGLFRFa (0.3 or 3.0 μg/g BW per peptide), or the same volume of mollusk saline (n = 12 per group). Spawning behavior was observed for 1 h after injection. Different letters indicate statistically significant differences (p < 0.05).

The spawned eggs from mature female abalone injected with GSLFRFa or GGLFRFa significantly (p < 0.05) increased in numbers compared with those of RFa-injected and saline-injected abalone (Fig 10A). In another experiment, both female and male abalone showed a significant (p < 0.05) increase in spawning behavior following injection of a high dose of GGLFRFa (3.0 μg/g BW) (Fig 10B and 10C).

## Discussion

Since the first invertebrate sNPF peptide in insects was discovered [17], sNPF and sNPF-related peptides have been widely identified in the protostomian phyla Mollusca, Annelida, Platyhelminthes, Arthropoda, and Nematoda [26]. Recently, a unique 22-aa-long vertebrate PrRP-type peptide was identified as a potent ligand for an sNPF/PrRP-type receptor in the starfish *Asterias rubens* [23], suggesting that sNPF-related peptides co-evolved with diversified sNPF receptors in the deuterostomian phylum Echinodermata. The invertebrate sNPF peptide sequences are distinct from the bilaterian NPF/Y sequences, which range in length from 18 to 40 aa with the C-terminal RXRF/Yamide motif [7, 26]. Here, we identified an LFRFa precursor including GSLFRFa, GGLFRFa, GTLFRFa, and GSTLFRFa in the Pacific abalone. GSLFRFa was previously identified as a functional ligand for Pacific oyster sNPFR [22]; however, the sequences of other LFRFa peptides were unknown. Our phylogenetic analysis further showed that the Hdh-LFRFa precursor is a sister member of the lophotrochozoan LFRFa/RYa/NPP and arthropod sNPF subfamilies, but branched separately from the bilaterian NPF/Y superfamily. Therefore, based on this study, molluscan LFRFa peptides are proposed to be orthologous to the well-known sNPF peptides, including not only RYa/NPP in phyla of the Lophotrochozoa but also sNPFs in phyla of the Nematoda, such as FLP15 and FLP18.

The sNPFRs and sNPFR-related receptors identified to date belong to the superfamily of rhodopsin-like GPCRs [33, 48, 49]. Likewise, the Hdh-sNPFR identified in this study harbors typical rhodopsin-like aa sequences in its seven TMDs: GN in TMD1, NLX_3_DX_8_P in TMD2, SX_3_L/SX_2_IX_2_DRY in TMD3, WX_8_P in TMD4, F/YX_2_PX_7_Y in TMD5, FX_3_WXP in TMD6, and SX_3_NPX_2_YX_6_F in TMD7 connected to the ICD amphipathic helix [50]. Interestingly, two notable aa changes (S and Y in TMD3 and TMD5, respectively) were found in the lophotrochozoan sNPFRs, including Pacific oyster sNPFR and *P*. *dumerili* NKY receptor, which are clearly different from the arthropod sNPFRs. However, the specific effects of these aa changes and post-translational modification sites on the structure, ligand binding, and downstream signaling of sNPFRs remain unclear.

Through the aa sequence comparison and phylogenetic analysis, we clarified that Hdh-sNPFR is positioned within a branch of the phylogenetic tree that comprises the lophotrochozoan and ecdysozoan sNPFR and sNPFR-related receptors, including *C*. *elegans* NPR1–5-type receptors. In other words, the protostome sNPFR subfamily is most likely a distinct receptor group from the bilaterian NPF/Y receptor superfamily. Consistent with this analysis, a recent phylogenetic study with novel sNPF/PrRP-type receptors in echinoderms revealed that all examined sNPFR-like sequences formed a monophyletic clade [23]. Thus, it has now become clear that the sNPF and NPF/Y signaling systems originated in the common ancestor of protostomes and deuterostomes [26]. However, the present study showed that the echinoderm sNPF/PrRP-type receptors were positioned in a clade comprising the deorphanized molluscan NPFRs [7], suggesting a receptor-sensitive classification of the sNPFR and NPF/Y superfamilies. One potential explanation for the difficulty in finding the endogenous sNPF and NPF/Y systems can be attributed to the substantial diversification in the canonical sequences of sNPF and NPF/Y peptides in echinoderms. In fact, a unique PrRP-like peptide was revealed as a potent ligand for the sNPF/PrRP-type receptor and the NPF/Y signaling system has reportedly been lost in deuterostome invertebrates [23].

Discovery of deorphanization of the bivalve Pacific oyster sNPFR was the first report on the functional characterization of molluscan sNPFR that is activated by endogenous LFRFa peptides [22]. The oyster LFRFa peptides could induce intracellular calcium mobilization, suggesting coupling of the oyster sNPFR to Gq-PKC signaling molecules. The present study is therefore the second such functional report in mollusks, demonstrating that LFRFa peptides are most likely the specific ligands for sNPFR in Pacific abalone. Hdh-LFRFa peptides also changed the subcellular localization of Hdh-sNPFR from the cell surface to the cytosolic compartment, suggesting that these peptides interact with Hdh-sNPFR on the cell membrane. The inhibitory mode of LFRFa peptides on forskolin-stimulated CRE-Luc activity indicates that the abalone LFRFa interaction with Hdh-sNPFR is involved in the cAMP/PKA signaling pathway through Gαi-subunit protein. This result is in line with the inhibitory activities of sNPF peptides on forskolin-stimulated cAMP accumulation observed in mammalian cells expressing insect sNPFRs derived from fire ant (*Solenopsis invicta*) and mosquito (*Anopheles gambiae*) [36, 51]. Collectively, these results indicate that molluscan LFRFa peptides are functional orthologs for arthropod sNPF peptides. In addition, we evaluated whether the Hdh-sNPFR is a Gq-coupled receptor using the SRE-Luc reporter, because Gq-coupled receptors can also activate SRE-mediated reporters via PKC-dependent mitogen-activated protein kinase signaling [52]. However, the abalone LFRFa peptides could not alter the SRE-Luc activity in the Hdh-sNPFR-expressing HEK293 cells, suggesting that the gastropod sNPFR mediates a different signaling pathway than that of bivalve sNPFR. In fact, endogenous coupling of sNPFRs with a Gq-subunit has been observed for other insect sNPFRs, such as those of the flies *D*. *melanogaster* and *Glossina morsitans* [34, 53]. Furthermore, dual cellular signaling mechanisms through Gq- and Gαi-subunit proteins were found in the sNPFR of desert locust (*Schistocerca gregaria*) [35]. The present study may provide further insight into the evolution of the sNPF signaling system in mollusks, based on the ligand–receptor co-evolution theory [54].

In invertebrates, transcripts for LFRFa/sNPF-like precursors and sNPFRs show higher expression levels in the neural ganglia and the brain than in the peripheral tissues [20, 25, 26]. Consistently, the *prepro-Hdh-LFRFa* and *Hdh-sNPFR* mRNAs were expressed at significantly higher levels in the CG and PPG than in other tissues examined in adult female abalone. We also detected significant increases of *prepro-Hdh-LFRFa* transcript levels in the ovary, intestine, and hepatopancreas in mature females compared with those of immature females, suggesting a role of the *Hdh-LFRFa* precursor gene in the reproductive and metabolic activities in female abalone. In other mollusks, LFRFa transcripts and peptides were detected in the ovary and in the oviduct nerve endings, supporting a mode of local peptide synthesis and release [22, 55, 56]. In Platyhelminthes, the expression of *NPP-4*, a putative ortholog of molluscan *LFRFa*, was enriched in regions of the cephalic ganglia but weakly detected in the post-pharyngeal copulatory apparatus [57]. Although the expression profile of molluscan *sNPFR* has only been examined in Pacific oyster to date [22], the *Hdh-sNPFR* expression pattern was similar to that of Pacific oyster, indicating a conserved expression pattern of molluscan *sNPFR* in the neural ganglia and the peripheral tissues along the reproductive cycle.

*The in vivo* injection experiment demonstrated that Hdh-LFRFa peptides decrease food consumption in Pacific abalone. The involvement of sNPF in feeding and metabolism has been demonstrated in many insects, although sNPF can act as either a stimulating or inhibiting factor depending on the examined species [26]. Along with insect sNPFs, LFRFa peptides convert feeding motor programs from ingestive to egestive, depress feeding muscle contractions in *Aplysia californica* [42], and increase rectal contractions in *Sepia officinalis* [55]. In addition, sNPFR expression was found to be upregulated in starved oysters, suggesting that LFRFa signaling is involved in feeding and metabolism in mollusks [22]. We recently reported that injection of NPF peptide increased food consumption in Pacific abalone, suggesting that NPF is an orexigenic neuropeptide in this species [7]. This observation is in contrast to the anorexic effect of Hdh-sNPF. Given the accumulated knowledge in insects that NPF and sNPF signaling systems are distinct and not likely to play redundant roles [25], the opposing effects of Hdh-NPF and Hdh-sNPF on food intake can be further explored to gain deeper insight into the functional evolution of neuropeptide systems in Lophotrochozoa.

Recent studies suggested the possibility of LFRFa-like signaling in reproduction regulation in mollusks [21, 26], although the direct activity of LFRFa peptides has not been investigated. Here, we found that injection of Hdh-LFRFa peptides increased the spawning behavior and egg numbers in mature abalone, indicating the potential role of LFRFa as a reproductive neuropeptide that stimulates spawning in mollusks. In fact, the Pacific oyster LFRFa peptides and *sNPFR* transcripts were detected in the majority of peripheral tissues examined, and the *sNPFR* expression level increased in the gonadic area when storage was maximal [22]. The cuttlefish LFRFa peptide was also detected in the nerve endings of the oviduct and nidamental gland [55, 56]. Similar to insect sNPFs, which are employed as a co-transmitter in neurons operating with neurotransmitters and regulate hormone release and vitellogenesis [25], both direct and indirect experimental data suggest a possible role of the LFRFa system in regulation of reproduction in mollusks.

Taken together, our results suggest that the Hdh-LFRFa/sNPFR system is most likely implicated in the regulation of energy metabolism and spawning in Pacific abalone. Although the LFRFa/sNPF-related signaling system remains to be fully elucidated with the accumulation of data from diverse invertebrate phyla, this study provides new insights into the distinct function and evolutionary history between the lophotrochozoan LFRFa and NPF systems. Our Hdh-sNPFR-based functional assay and molecular data will enable further characterization of neuropeptides and orphan GPCRs to better understand their functions *in vitro* and *in vivo*.

## Supporting information

S1 Fig Phylogenetic tree based on amino acid sequences of LFRFa/sNPF/NPF-related precursors. Hdh-APGWa and *L*.*sta*_APGWa precursors were used as an outgroup. The trimmed amino acid sequences were used for each neuropeptide precursor (see S1 Table) and the maximum-likelihood tree was generated using W-IQ server v1.6.12. Bootstrap values are given at each branch. The scale bar indicates amino acid substitutions per site.
(TIF)

S2 Fig Alignment of invertebrate LFRFa/sNPF-related peptide sequences. Selected LFRFa, sNPF, and LFRFa/sNPF-related peptides (see S1 Table) aligned using Clustal Omega Multiple Sequence Alignment with default parameters. Identical and highly conserved residues (>70%) are shaded in black and gray, respectively. Blue and underlined amino acid sequences indicate deorphanized peptides and highly reactive peptides compared with those of the same species, respectively.
(TIF)

S1 Table Sources and accession numbers of the LFRFa/sNPF-related precursor sequences used for the phylogenetic analysis shown in Fig 2. (DOCX)

S2 Table Sources and accession numbers of the receptor sequences used for the phylogenetic analysis and sequence alignment shown in Figs 3 and 4. (DOCX)

S3 Table List of reference genes and validation data for RT-qPCR. (DOCX)

## References

[1] Ponder WF, Lindberg DR. Phylogeny and evolution of the mollusca. Berkeley: University of California Press; 2008.

[2] Cummins SF, Tollenaere A, Degnan BM, Croll RP. Molecular analysis of two FMRFamide-encoding transcripts expressed during the development of the tropical abalone *Haliotis asinina*. J Comp Neurol. 2011;519: 2043–2059. doi: 10.1002/cne.22621 21452226

[3] Cook PA, Gordon HR. World abalone supply, markets, and pricing. J Shellfish Res. 2010;29: 569–571.

[4] Park CJ, Kim SY. Abalone aquaculture in Korea. J Shellfish Res. 2013;32: 17–19.

[5] Kim MA, Rhee JS, Kim TH, Lee JS, Choi AY, Choi BS, et al. Alternative splicing profile and sex-preferential gene expression in the female and male Pacific abalone *Haliotis discus hannai*. Genes 2017;8: 99. doi: 10.3390/genes8030099 28282934 PMC5368703

[6] Kim MA, Markkandan K, Han NY, Park JM, Lee JS, Lee H, et al. Neural ganglia transcriptome and peptidome associated with sexual maturation in female Pacific abalone (*Haliotis discus hannai*). Genes 2019;10: 268. 10.3390/genes10040268 PMC6523705 30987054

[7] Kim KS, Kim MA, Park K, Sohn YC. NPF activates a specific NPF receptor and regulates food intake in Pacific abalone *Haliotis discus hannai*. Sci Rep. 2021;11: 20912. doi: 10.1038/s41598-021-00238-1 34686694 PMC8536682

[8] Vanden Broeck J. Neuropeptides and their precursors in the fruitfly, *Drosophila melanogaster*. Peptides 2001;22: 241–254. doi: 10.1016/s0196-9781(00)00376-4 11179818

[9] De Haes W, Van Sinay E, Detienne G, Temmerman L, Schoofs L, Boonen K. Functional neuropeptidomics in invertebrates. Biochim Biophys Acta 2015;1854: 812–826. doi: 10.1016/j.bbapap.2014.12.011 25528324

[10] Hewes RS, Taghert PH. Neuropeptides and neuropeptide receptors in the *Drosophila melanogaster* genome. Genome Res. 2001;11: 1126–1142. doi: 10.1101/gr.169901 11381038 PMC311076

[11] Bendena WG, Campbell J, Zara L, Tobe SS, Chin-Sang ID. Select neuropeptides and their G-protein coupled receptors in *Caenorhabditis elegans* and *Drosophila melanogaster*. Front Endocrinol. 2012;3: 93. doi: 10.3389/fendo.2012.00093 22908006 PMC3414713

[12] Predel R, Neupert S, Garczynski SF, Crim JW, Brown MR, Russell WK, et al. Neuropeptidomics of the mosquito *Aedes aegypti*. J Proteome Res. 2010;9: 2006–2015. doi: 10.1021/pr901187p 20163154 PMC2853887

[13] van Wielendaele P, Badisco L, Vanden Broeck J. Neuropeptidergic regulation of reproduction in insects. Gen Comp Endocrinol. 2013;188: 23–34. doi: 10.1016/j.ygcen.2013.02.005 23454669

[14] Schoofs L, De Loof A, Van Hiel MB. Neuropeptides as regulators of behavior in insects. Ann Rev Entomol. 2017;62: 35–52. doi: 10.1146/annurev-ento-031616-035500 27813667

[15] Taghert PH, Nitabach MN. Peptide neuromodulation in invertebrate model systems. Neuron 2012;76: 82–97. doi: 10.1016/j.neuron.2012.08.035 23040808 PMC3466441

[16] Wettschureck N, Offermanns S. Mammalian G proteins and their cell type specific functions. Physiol Rev. 2005;85: 1159–1204. doi: 10.1152/physrev.00003.2005 16183910

[17] Semmens DC, Mirabeau O, Moghul I, Pancholi MR, Wurm Y, Elphick MR. Transcriptomic identification of starfish neuropeptide precursors yields new insights into neuropeptide evolution. Open Biol. 2016;6: 150224. doi: 10.1098/rsob.150224 26865025 PMC4772807

[18] Elphick MR, Mirabeau O, Larhammar D. Evolution of neuropeptide signalling systems. J Exp Biol. 2018;221: jeb151092. doi: 10.1242/jeb.151092 29440283 PMC5818035

[19] Price DA, Greenberg MJ. Structure of a molluscan cardioexcitatory neuropeptide. Science 1977;197: 670–671. doi: 10.1126/science.877582 877582

[20] Walker RJ, Papaioannou S, Holden-Dye L (2009) A review of FMRFamide- and RFamide-like peptides in metazoa. Invert Neurosci. 9: 111–153. doi: 10.1007/s10158-010-0097-7 20191373

[21] Zatylny-Gaudin C, Favrel P. Diversity of the RFamide peptide family in mollusks. Front Endocrinol. 2014;5: 178. doi: 10.3389/fendo.2014.00178 25386166 PMC4208409

[22] Bigot L, Beets I, Dubos MP, Boudry P, Schoofs L, Favrel P. et al. Functional characterization of a short neuropeptide F-related receptor in a lophotrochozoan, the mollusk *Crassostrea gigas*. J Exp Biol. 2014;217: 2974–2982. doi: 10.1242/jeb.104067 24948637

[23] Yañez-Guerra LA, Zhong X, Moghul I, Butts T, Zampronio CG, Jones AM, et al. Echinoderms provide missing link in the evolution of PrRP/sNPF-type neuropeptide signalling. Elife 2020;9: e57640. doi: 10.7554/eLife.57640 32579512 PMC7314547

[24] Veenstra JA, Lambrou G. Isolation of a novel RFamide peptide from the midgut of the American cockroach, *Periplaneta americana*. Biochem Biophys Res Commun. 1995;213: 519–524. doi: 10.1006/bbrc.1995.2162 7646507

[25] Nässel DR, Wegener C. A comparative review of short and long neuropeptide F signaling in invertebrates: Any similarities to vertebrate neuropeptide Y signaling? Peptides 2011; 32: 1335–1355. doi: 10.1016/j.peptides.2011.03.013 21440021

[26] Fadda M, Hasakiogullari I, Temmerman L, Beets I, Zels S, Schoofs L. Regulation of feeding and metabolism by neuropeptide F and short neuropeptide F in invertebrates. Front Endocrinol. 2019;10: 64. doi: 10.3389/fendo.2019.00064 30837946 PMC6389622

[27] Mirabeau O, Joly JS. Molecular evolution of peptidergic signaling systems in bilaterians. Proc Natl Acad Sci USA. 2013; 110: E2028–E2037. doi: 10.1073/pnas.1219956110 23671109 PMC3670399

[28] Lagerström MC, Fredriksson R, Bjarnadóttir TK, Fridmanis D, Holmquist T, Andersson J, et al. Origin of the prolactin-releasing hormone (PRLH) receptors: evidence of coevolution between PRLH and a redundant neuropeptide Y receptor during vertebrate evolution. Genomics 2005; 85: 688–703. doi: 10.1016/j.ygeno.2005.02.007 15885496

[29] Lee KS, You KH, Choo JK, Han YM, Yu K. Drosophila short neuropeptide F regulates food intake and body size. J Biol Chem. 2004;279: 50781–50789. doi: 10.1074/jbc.M407842200 15385546

[30] Lee KS, Kwon OY, Lee JH, Kwon K, Min KJ, Jung SA, et al. Drosophila short neuropeptide F signalling regulates growth by ERK-mediated insulin signalling. Nat Cell Biol. 2008;10: 468–475. doi: 10.1038/ncb1710 18344986

[31] Kahsai L, Kapan N, Dircksen H, Winther AM, Nässel DR. Metabolic stress responses in Drosophila are modulated by brain neurosecretory cells that produce multiple neuropeptides. PLoS One 2010;5: e11480. doi: 10.1371/journal.pone.0011480 20628603 PMC2900207

[32] Kahsai L, Martin JR, Winther AM. Neuropeptides in the Drosophila central complex in modulation of locomotor behavior. J Exp Biol. 2010;213: 2256–2265. doi: 10.1242/jeb.043190 20543124

[33] Bao C, Yang Y, Huang H, Ye H. Inhibitory role of the mud crab short neuropeptide F in vitellogenesis and oocyte maturation via autocrine/paracrine signaling. Front Endocrinol. 2018;9: 390. doi: 10.3389/fendo.2018.00390 30057569 PMC6053504

[34] Mertens I, Meeusen T, Huybrechts R, De Loof A, Schoofs L. Characterization of the short neuropeptide F receptor from *Drosophila melanogaster*. Biochem Biophys Res Commun. 2002;297: 1140–1148. doi: 10.1016/s0006-291x(02)02351-3 12372405

[35] Dillen S, Zels S, Verlinden H, Spit J, Van Wielendaele P, Broeck JV. et al. Functional characterization of the short neuropeptide F receptor in the desert locust, Schistocerca gregaria. PLoS One 2013;8: e53604. doi: 10.1371/journal.pone.0053604 23308260 PMC3537624

[36] Bajracharya P, Lu HL, Pietrantonio PV. The red imported fire ant (*Solenopsis invicta* Buren) kept Y not F: predicted sNPY endogenous ligands deorphanize the short NPF (sNPF) receptor. PLoS One 2014;9: e109590. doi: 10.1371/journal.pone.0109590 25310341 PMC4195672

[37] Madeira F, Park YM, Lee J, Buso N, Gur T, Madhusoodanan N, et al. The EMBL-EBI search and sequence analysis tools APIs in 2019. Nucleic Acids Res. 2019;47: W636–W641. doi: 10.1093/nar/gkz268 30976793 PMC6602479

[38] Krogh A, Larsson B, von Heijne G, Sonnhammer EL. Predicting transmembrane protein topology with a hidden Markov model: application to complete genomes. J Mol Biol. 2001;305: 567–580. doi: 10.1006/jmbi.2000.4315 11152613

[39] Fadda M, De Fruyt N, Borghgraef C, Watteyne J, Peymen K, Vandewyer E, et al. NPY/NPF-related neuropeptide FLP-34 signals from serotonergic neurons to modulate aversive olfactory learning in *Caenorhabditis elegans*. J Neurosci. 2020;40: 6018–6034. doi: 10.1523/JNEUROSCI.2674-19.2020 32576621 PMC7392509

[40] Thiel D, Yañez-Guerra LA, Franz-Wachtel M, Hejnol A, Jékely G. Nemertean, brachiopod, and phoronid neuropeptidomics reveals ancestral spiralian signaling systems. Mol Biol Evol. 2021;38: 4847–4866. doi: 10.1093/molbev/msab211 34272863 PMC8557429

[41] Trifinopoulos J, Nguyen LT, von Haeseler A, Minh BQ. W-IQ-TREE: a fast online phylogenetic tool for maximum likelihood analysis. Nucleic Acids Res. 2016;44: W232–W235. doi: 10.1093/nar/gkw256 27084950 PMC4987875

[42] Vilim FS, Sasaki K, Rybak J, Alexeeva V, Cropper EC, Jing J, et al. Distinct mechanisms produce functionally complementary actions of neuropeptides that are structurally related but derived from different precursors. J Neurosci. 2010;30: 131–147. doi: 10.1523/JNEUROSCI.3282-09.2010 20053896 PMC2826173

[43] Kim KS, Kim MA, Sohn YC. Molecular characterization, expression analysis, and functional properties of multiple 5-hydroxytryptamine receptors in Pacific abalone (*Haliotis discus hannai*). Gen Comp Endocrinol. 2019;276: 52–59. doi: 10.1016/j.ygcen.2019.03.001 30849410

[44] Kim TH, Kim MA, Kim KS, Kim JW, Lim HK, Lee JS, et al. Characterization and spatiotemporal expression of gonadotropin-releasing hormone in the Pacific abalone, *Haliotis discus hannai*. Comp Biochem Physiol A Mol Integr Physiol. 2017;209: 1–9. doi: 10.1016/j.cbpa.2017.04.001 28408352

[45] Sundaram VK, Sampathkumar NK, Massaad C, Grenier J. Optimal use of statistical methods to validate reference gene stability in longitudinal studies. PLoS One 2019;14: e0219440. doi: 10.1371/journal.pone.0219440 31335863 PMC6650036

[46] Kim KS, Kim TH, Kim MA, Lee JS, Sohn YC. Expression profile and reproductive regulation of APGWamide in Pacific abalone (Haliotis discus hannai). Comp Biochem Physiol A Mol Integr Physiol. 2018;222: 26–35. doi: 10.1016/j.cbpa.2018.04.005 29679684

[47] Gether U. Uncovering molecular mechanisms involved in activation of G protein-coupled receptors. Endocr Rev. 2000;21: 90–113. doi: 10.1210/edrv.21.1.0390 10696571

[48] Feng G, Reale V, Chatwin H, Kennedy K, Venard R, Ericsson C, et al. Functional characterization of a neuropeptide F-like receptor from *Drosophila melanogaster*. Eur J Neurosci. 2003;18: 227–238. doi: 10.1046/j.1460-9568.2003.02719.x 12887405

[49] Jekely G. Global view of the evolution and diversity of metazoan neuropeptide signaling. Proc Natl Acad Sci USA. 2013;110: 8702–8707. doi: 10.1073/pnas.1221833110 23637342 PMC3666674

[50] Costanzi S. Homology modeling of class a G protein-coupled receptors. Methods Mol Biol. 2012; 857: 259–279. doi: 10.1007/978-1-61779-588-6_11 22323225 PMC3354613

[51] Garczynski SF, Crim JW, Brown MR. Characterization and expression of the short neuropeptide F receptor in the African malaria mosquito, *Anopheles gambiae*. Peptides 2007;28: 109–118. doi: 10.1016/j.peptides.2006.09.019 17140700 PMC1864938

[52] Hill SJ, Baker JG, Rees S. Reporter-gene systems for the study of G-protein-coupled receptors. Curr Opin Pharmacol. 2001;1: 526–532. doi: 10.1016/s1471-4892(01)00091-1 11764780

[53] Caers J, Peymen K, Van Hiel MB, Van Rompay L, Van Den Abbeele J, Schoofs L, et al. Molecular characterization of a short neuropeptide F signaling system in the tsetse fly, *Glossina morsitans*. Gen Comp Endocrinol. 2016;235: 142–149. doi: 10.1016/j.ygcen.2016.06.005 27288635

[54] van Kesteren RE, Tensen CP, Smit AB, van Minnen J, Kolakowski LF, Meyerhof W, et al. Co-evolution of ligand-receptor pairs in the vasopressin/oxytocin superfamily of bioactive peptides. J Biol Chem. 1996;271: 3619–3226. doi: 10.1074/jbc.271.7.3619 8631971

[55] Zatylny-Gaudin C, Bernay B, Zanuttini B, Leprince J, Vaudry H, Henry J. Characterization of a novel LFRFamide neuropeptide in the cephalopod *Sepia officinalis*. Peptides 2010;31: 207–214. doi: 10.1016/j.peptides.2009.11.021 19954756

[56] Cao ZH, Sun LL, Chi CF, Liu HH, Zhou LQ, Lv ZM, et al. Molecular cloning, expression analysis and cellular localization of an LFRFamide gene in the cuttlefish *Sepiella japonica*. Peptides 2016;80: 40–47. doi: 10.1016/j.peptides.2015.10.005 26494614

[57] Collins JJ 3rd, Hou X, Romanova EV, Lambrus BG, Miller CM, Saberi A, et al. Genome-wide analyses reveal a role for peptide hormones in planarian germline development. PLoS Biol. 2010;8: e1000509. doi: 10.1371/journal.pbio.1000509 20967238 PMC2953531

