# Supplementary figures and images for: Functional analysis of LFRFamide signaling in Pacific abalone, Haliotis discus hannai
Source: PLoS One. 2022 May 5;17(5):e0267039. doi: 10.1371/journal.pone.0267039 (PMC9071130; doi:10.1371/journal.pone.0267039)

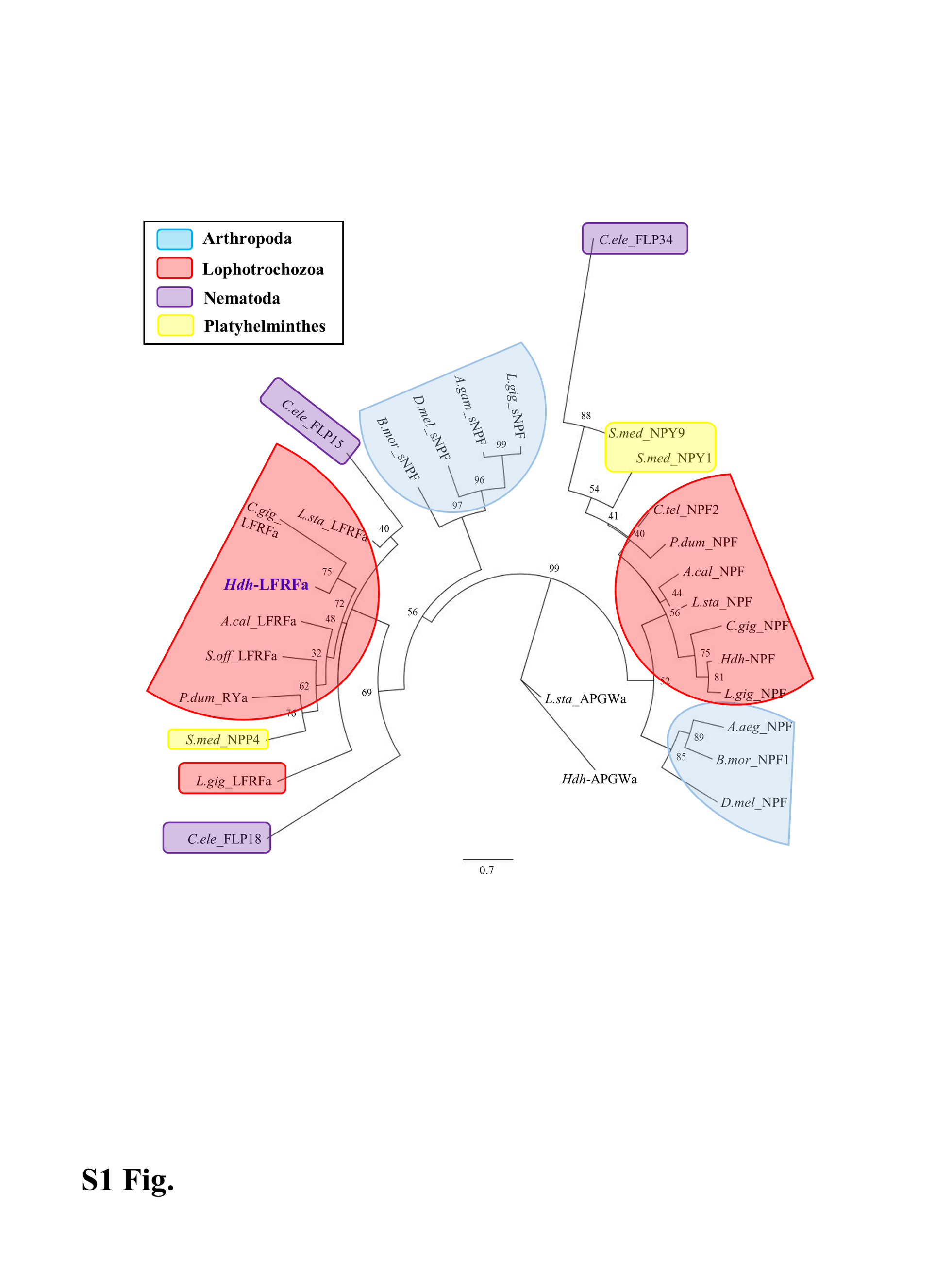

Supplement: S1 Fig — Hdh-APGWa and L.sta_APGWa precursors were used as an outgroup. The trimmed amino acid sequences were used for each neuropeptide precursor (see S1 Table) and the maximum-likelihood tree was generated using W-IQ server v1.6.12. Bootstrap values are given at each branch. The scale bar indicates amino acid substitutions per site. (TIF) [file pone.0267039.s001.tif]

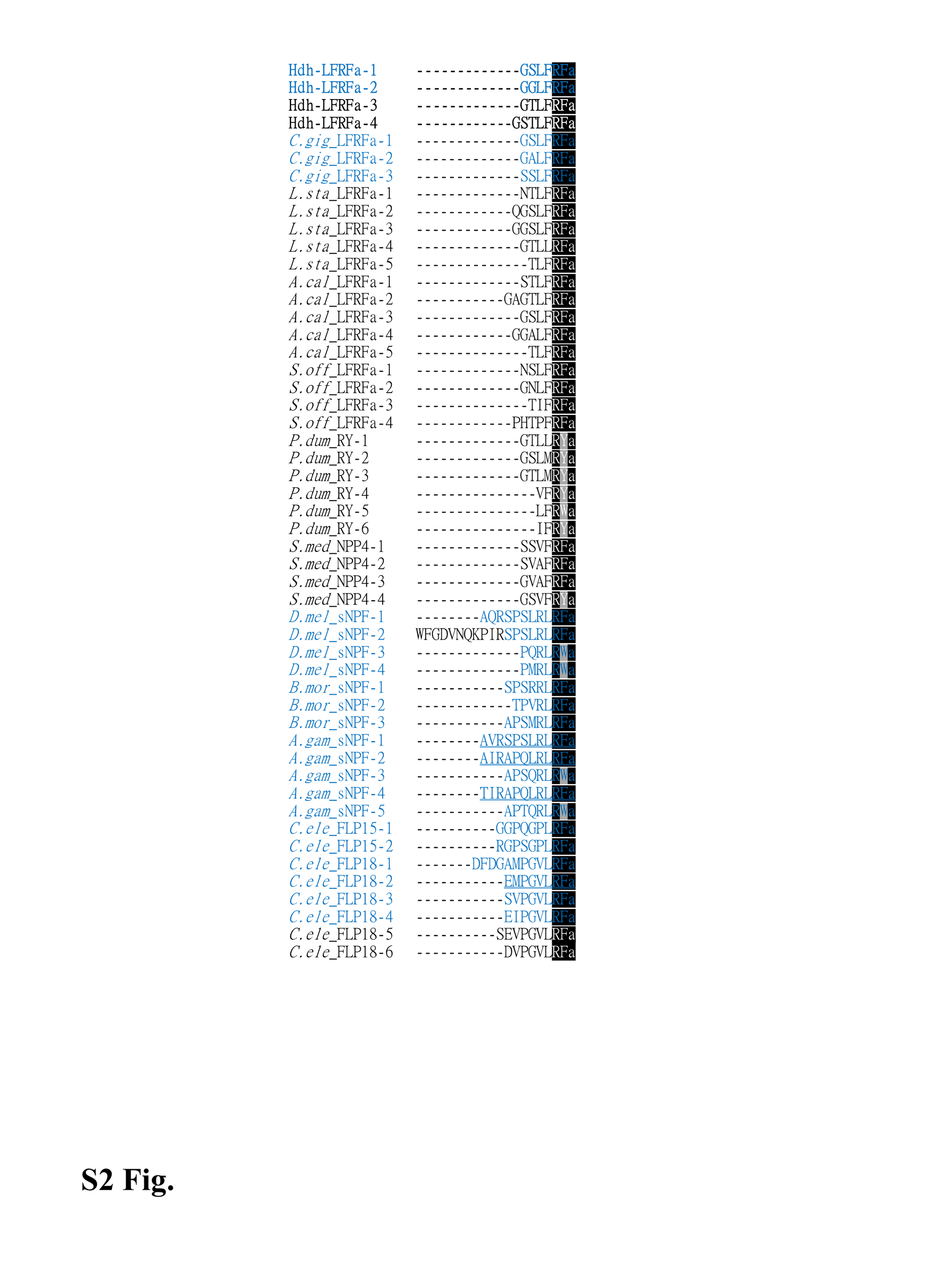

Supplement: S2 Fig — Selected LFRFa, sNPF, and LFRFa/sNPF-related peptides (see S1 Table) aligned using Clustal Omega Multiple Sequence Alignment with default parameters. Identical and highly conserved residues (>70%) are shaded in black and gray, respectively. Blue and underlined amino acid sequences indicate deorphanized peptides and highly reactive peptides compared with those of the same species, respectively. (TIF) [file pone.0267039.s002.tif]
