# Supplementary material for: Functional analysis of LFRFamide signaling in Pacific abalone, Haliotis discus hannai
Source: PLoS One. 2022 May 5;17(5):e0267039. doi: 10.1371/journal.pone.0267039 (PMC9071130; doi:10.1371/journal.pone.0267039)
Supplement: S1 Table — (DOCX) [file pone.0267039.s003.docx]

**S1 Table.** Sources and accession numbers of the LFRFa/sNPF-related precursor sequences used for the phylogenetic analysis shown in Figure 2.

| **Sequence Name** | **Original name** | **Species** | **Accession numbers** |
| --- | --- | --- | --- |
| *Hdh*-LFRFa | LFRFa precursor | *Haliotis discus hannai* | OL804262 |
| *C.gig*_LFRFa | LFRFa precursor | *Crassostrea_gigas* | EKC33711 |
| *L.sta*_LFRFa | LFRFa precursor | *Lymnaea stagnalis* | AY773478.1 |
| *A.cal_*LFRFa | FRFa precursor | *Aplysia_californica* | NP_001191429.1 |
| *L.gig*_LFRFa | LFRFa precursor | *Lottia gigantea* | - |
| *S.off*_LFRFa | FMRFa-related peptide 2 | *Sepia officinalis*  *Sepiella japonica* | GU388435.1  KP260902.1 |
| *P.dum*_RYa | RYa precursor | *Platynereis dumerili* | JF811330.1 |
| *S.med_*NPP4 | Neuropeptide precursor 4 | *Schmidtea mediterranea* | BK007037.1 |
| *D.mel*_sNPF | sNPF | *Drosophila melanogaster* | NM_165316.2 |
| *B.mor*_sNPF | sNPF | *Bombyx mori* | AB330419.1 |
| *A.gam_*sNPF | sNPF | *Anopheles gambiae* | DQ437578.1 |
| *C.ele*_FLP15 | FMRFa-related peptide 15 | *Caenorhabditis elegans* | NP_499820.1 |
| *C.ele*_FLP18 | FMRFa-related peptide 18 | *Caenorhabditis elegans* | NP_508514.2 |
| *Hdh*-NPF | NPF precursor | *Haliotis discus hannai* | MZ027150 |
| *L.sta*_NPF | NPF precursor | *Lymnaea stagnalis* | AJ238276.1 |
| *Hdh*-APGWa | APGWa precursor | *Haliotis discus hannai* | MG751779.1 |
| *L.sta*_APGWa | APGWa precursor | *Lymnaea stagnalis* | 1811269A |
