# Supplementary material for: Functional analysis of LFRFamide signaling in Pacific abalone, Haliotis discus hannai
Source: PLoS One. 2022 May 5;17(5):e0267039. doi: 10.1371/journal.pone.0267039 (PMC9071130; doi:10.1371/journal.pone.0267039)
Supplement: S2 Table — (DOCX) [file pone.0267039.s004.docx]

**S2 Table.** Sources and accession numbers of the receptor sequences used for the phylogenetic analysis and the sequence alignment as shown in Figures 3 and 4.

| **Sequence Name** | **Species** | **Accession number** |
| --- | --- | --- |
| *Hdh*-sNPFR | *Haliotis discus hannai* | OL907301 |
| *Hdh*-NPFR | *Haliotis discus hannai* | MZ014382 |
| *Hdh*-NPFR-like-1 | *Haliotis discus hannai* | MZ014383 |
| *Hdh*-NPFR-like-2 | *Haliotis discus hannai* | MZ014384 |
| *Hdh*-NPFR-like-3 | *Haliotis discus hannai* | MZ014385 |
| *A.cal*_NPYR2 | *Aplysia_californica* | XP_005089627.1 |
| *A.cal*_NPFR-like | *Aplysia_californica* | XP_005089880.1 |
| *C.gig*_NPFR-like | *Crassostrea_gigas* | XP_011444490.1 |
| *L.gig*_NPFR | *Lottia_gigantea* | XP_009066442.1 |
| *T.cas*_NPFR | *Tribolium_castaneum* | XP_008198436.1 |
| *D.mel*_NPFR | *Drosophila melanogaster* | NP_001246947.1 |
| *A.aeg*_NPFR | *Aedes_aegypti* | XP_021693392.1 |
| *C.tel*_NPFR-like | *Capitella teleta* | ELT88377.1 |
| *C.ele*_NPR-12 | *Caenorhabditis elegans* | NP_001293732.1 |
| *C.ele*_NPR-11 | *Caenorhabditis elegans* | NP_508234.2 |
| *S.med*_NPYR1 | *Schmidtea mediterranea* | ANO39130.1 |
| *S.med*_NPYR5 | *Schmidtea mediterranea* | ANO39140.1 |
| *C.tel*_NPY/NPF-R | *Capitella teleta* | ELT98787.1 |
| *P.dum*_GPCR62 | *Platynereis_dumerili* | AKQ63068.1 |
| *G.gal*_NPYR1 | *Gallus gallus* | AAK83557.1 |
| *H.sap*_NPYR1 | *Homo sapiens* | NP_000900.1 |
| *G.gal*_NPYR4 | *Gallus gallus* | AAL84161.1 |
| *H.sap*_NPYR4 | *Homo sapiens* | NP_005963.4 |
| *D.rer*_NPYR8a | *Danio rerio* | NP_571512.1 |
| *D.rer*_NPYR8b | *Danio rerio* | NP_571511.1 |
| *D.rer*_NPYR4 | *Danio rerio* | NP_571515.1 |
| *G.gal*_NPYR5 | *Gallus gallus* | AAK83556.1 |
| *H.sap*_NPYR5 | *Homo sapiens* | NP_006165.1 |
| *D.rer*_NPYR2 | *Danio rerio* | XP_001343301.3 |
| *X.tro*_NPYR2 | *Xenopus tropicalis* | XP_004911210.1 |
| *G.gal*_NPYR2 | *Gallus gallus* | NP_001026299.1 |
| *H.sap*_NPYR2 | *Homo sapiens* | NP_000901.1 |
| *G.gal_*NPYR7 | *Gallus gallus* | NP_001032913.1 |
| *D.rer_*NPYR7 | *Danio rerio* | NP_001007219.1 |
| *L.sta_*GRL105 | *Lymnaea stagnalis* | CAA57620.1 |
| *A.rub*_sNPF/PrRP-R | *Asterias rubens* | AYM55328.1 |
| *S.pur*_sNPFR/PrRP-R | *Strongylocentrotus purpuratus* | XP_003725178.2 |
| *C.gig*_sNPFR | *Crassostrea gigas* | XP_011451552.1 |
| *S.gre*_sNPFR | *Schistocerca gregaria* | AGC54822.1 |
| *C.tel*_sNPFR | *Capitella teleta* | ELT88594.1 |
| *P.dum_*NKY-R | *Platynereis dumerilii* | AKQ63001.1 |
| *F.bus_*NPYR | *Fasciolopsis buski* | KAA0185570.1 |
| *P.xen_*NPYR | *Protopolystoma xenopodis* | VEL36810 |
| *G.aeg_*PrRP-R | *Gigantopelta aegis* | XP_041371228.1 |
| *D.mel*_sNPFR-A | *Drosophila melanogaster* | NP_524176.1 |
| *B.mor*_sNPFR(GPR-A7) | *Bombyx_mori* | NP_001127742.1 |
| *B.mor*_sNPFR(GPR-A11) | *Bombyx_mori* | NP_001127708.1 |
| *B.mor*_sNPFR(GPR-A10) | *Bombyx_mori* | NP_001127707.1 |
| *A.aeg*_sNPFR | *Aedes aegypti* | AGX84998.1 |
| *C.ele*_NPR5 | *Caenorhabditis elegans* | CCD70460.1 |
| *C.ele*_NPR4 | *Caenorhabditis elegans* | NP_001300304.1 |
| *C.ele*_NPR3 | *Caenorhabditis elegans* | CAB05681.1 |
| *C.ele*_NPR2 | *Caenorhabditis elegans* | NP_501701.2 |
| *C.ele*_NPR1 | *Caenorhabditis elegans* | NP_508816.1 |
| *B.flo*_PrRP-R | *Branchiostoma floridae* | XP_002608333.1 |
| *H.sap*_PrRP-R | *Homo sapiens* | NP_004239.1 |
| *D.rer*-PrRP-R | *Danio rerio* | NP_001034615.1 |
| *G.gal*_PrRP-R | *Gallus gallus* | AAW30382.1 |
| *X.tro*_PrRP-R | *Xenopus tropicalis* | XP_002940396.1 |
| *S.kow*_PrRP-R1 | *Saccoglossus kowalevskii* | XP_002740053.1 |
| *S.kow*_PrRP-R2 | *Saccoglossus kowalevskii* | XP_006815575.1 |
| *S.kow*_PrRP-R3 | *Saccoglossus kowalevskii* | XP_002738225.1 |
| *C.ele*_Luqin-R | *Caenorhabditis elegans* | NP_001023541.1 |
| *A.pis*_RYamide-R1 | *Acyrthosiphon pisum* | XP_008178727.1 |
| *D.mel*_RYamide-R | *Drosophila melanogaster* | P25931.2 |
| *A.aeg*_RYamide-R | *Aedes aegypti* | AGX85003.1 |
| *A.cal*_Luqin-R | *Aplysia californica* | XP_012937781.1 |
| *L.sta*_Luqin-R | *Lymnaea stagnalis* | AAB92258.1 |
| *O.bim*_Luqin-R | *Octopus bimaculoides* | XP_014786450.1 |
| *C.tel*_Luqin-R | *Capitella teleta* | ELT96089.1 |
| *P.dum*_Luqin-R | *Platynereis dumerilii* | KP420214.1 |
| *S.pur*_RYamide-R | *Strongylocentrotus purpuratus* | XP_783326.1 |
| *A.rub*_Luqin-R1 | *Asterias rubens* | MG744509 |
| *S.kow*_Luqin-R1 | *Saccoglossus kowalevskii* | XM_002731957.1 |
| *S.kow*_Luqin-R2 | *Saccoglossus kowalevskii* | XM_002731958.1 |
| *L.gig*_Luqin-R1 | *Lottia_gigantea* | XP_009064514.1 |
| *L.gig*_Luqin-R2 | *Lottia_gigantea* | XP_009064591.1 |
| *C.int*_Tachykinin-R | *Ciona intestinalis* | XM_009863501.2 |
| *H.sap*_NK1R-SPR | *Homo sapiens* | NP_001049.1 |
| *H.sap*_NK2R | *Homo sapiens* | AAB20303.1 |
| *H.sap*_Neuromedin-K-R | *Homo sapiens* | NP_001050.1 |
| *D.mel*_Tachykinin-R | *Drosophila melanogaster* | FBtr0085507 |
| *O.vul*_Tachykinin-R | *Octopus vulgaris* | BAD93354.1 |
| *A.cal*_Tachykinin-R | *Aplysia californica* | XP_012936180.1 |
| *L.gig*_Tachykinin-R | *Lottia gigantea* | XP_009062052.1 |
| *S.pur*_Tachykinin-R | *Strongylocentrotus purpuratus* | XP_011662258.1 |
| *A.rub*_Tachykinin-R1 | *Asterias rubens* | XP_033644232.1 |
| *A.rub*_Tachykinin-R2 | *Asterias rubens* | MG744512.1 |
| *S.pur*_GPR83 | *Strongylocentrotus purpuratus* | XP_003729750.1 |
| *H.sap*_GPR83 | *Homo sapiens* | NP_057624.3 |
| *G.gal*_GPR83 | *Gallus gallus* | AEO92092.1 |
