# Supplementary material for: Functional analysis of LFRFamide signaling in Pacific abalone, Haliotis discus hannai
Source: PLoS One. 2022 May 5;17(5):e0267039. doi: 10.1371/journal.pone.0267039 (PMC9071130; doi:10.1371/journal.pone.0267039)
Supplement: S3 Table — (DOCX) [file pone.0267039.s005.docx]

| **S3 Table.** List of reference genes and validation data for RT-qPCR. | | | | | |
| --- | --- | --- | --- | --- | --- |
| Symbol | Gene name | Sequence (5' → 3') | Coeffecient  of variation (CV%) | Efficiencies (E%) | Regression coefficients (R^2^) |
| *RPL5* | Ribosomal protein L5 | F: TCACCAACAAGGACATCATTTGTC R: CAGGAGGAGTCCAGTGCAGTATG | 43.69 | 98.8 | 0.9966 |
| *CY* | Cyclophilin | F: GATCCAAGGTGGAGACTTCACTAAG R: AACTGGGAACCATTGGTGTTG | 53.91 | 101.41 | 0.9973 |
| *UBC* | Ubiquitin-conjuction enzyme | F: CACTGGCAAGCAACAATAATGG  R: CCATTGCTGTTGATGTTTGGA | 84.90 | - | - |
| *ELF* | Elongation factor 1-alpha | F: GGAAAACCCCAAAAGTGTCAAGT R: GTGGTGGGTATGAGGAGAAAGC | 68.45 | - | - |

*Note.* F, forward primer; R, reverse primer
